# Assessment of Trophoblast cell surface antigen 2 (Trop-2) expression and its clinical significance in breast cancer: a multi-level analysis of protein and gene expression

**DOI:** 10.1186/s12916-026-05093-3

**Published:** 2026-07-31

**Authors:** Maria Angeliki Toli, Michail Sarafidis, Panagiotis Filis, Evangelos Tzoras, Nikolaos Tsiknakis, Emmanouil Sifakis, Efstathia Liatsou, Georgios Rassidakis, Jonas Bergh, Alexios Matikas, Ioannis Zerdes, Theodoros Foukakis

**Affiliations:** 1 Department of Oncology and Pathology, Karolinska Institutet, Stockholm, Sweden; 2 Department of Hygiene and Epidemiology, School of Medicine, University of Ioannina, Ioannina, Greece; 3 Department of Surgery, Sahlgrenska University Hospital, Gothenburg, Sweden; 4 Department of Research, Development, Education and Innovation (FOUUI), Sahlgrenska University Hospital, Gothenburg, Sweden; 5 Department of Clinical Pathology and Cancer Diagnostics, Karolinska University Hospital, Stockholm, Sweden; 6 Department of Hematopathology, The University of Texas MD Anderson Cancer Center, Houston, TX USA; 7 Breast Center, Karolinska Comprehensive Cancer Center, Karolinska University Hospital, Stockholm, Sweden; 8 Theme Cancer, Karolinska Comprehensive Cancer Center, Karolinska University Hospital, Stockholm, Sweden

**Keywords:** Breast cancer, Biomarkers, Trop-2, Antibody-drug conjugates, Prognosis, Therapeutic target, Molecular target

## Abstract

**Background:**

Trophoblast cell surface antigen 2 (Trop-2) is a targetable transmembrane glycoprotein commonly overexpressed in breast cancer (BC). This study combines a systematic review and meta-analysis with a retrospective analysis of an independent early BC patient cohort, aiming to investigate the expression patterns of Trop-2 and their clinical significance in BC.

**Methods:**

A systematic literature search was conducted up to October 2025 to identify studies evaluating Trop-2 protein expression levels and clinical outcomes in patients with BC receiving Trop-2–directed antibody-drug conjugates (ADCs) or chemotherapy. Random-effects meta-analyses were performed to estimate pooled Trop-2 positivity rates and to assess progression-free (PFS) and overall survival (OS) across Trop-2 expression levels. Additionally, an association analysis between Trop-2 protein and gene expression was performed in an early BC patient cohort (n = 564) using immunohistochemistry and gene expression data. Trop-2 protein expression was assessed using both conventional observer-based methods and digital quantitative image analysis.

**Results:**

A total of 41 studies fulfilled the inclusion criteria. The overall pooled Trop-2 protein positivity rate was 72% [95% Confidence Interval (CI), 67–77%; 41 studies, n = 9170]. Trop-2–directed ADCs improved PFS versus chemotherapy across different H-score groups, with the greatest benefit in tumours with high H-score [3 studies; n = 575; Hazard Ratio (HR) = 0.33, 95% CI 0.20–0.56, p < 0.0001]. For OS, the benefit was observed in the low H-score group (2 studies; n = 272; HR = 0.75, 95% CI 0.57–0.98, p = 0.034). In the study cohort, Trop-2 protein by observer-read assessment was detected in 396/454 patients (87.22%). Digital pathology identified 54 (11.90%) tumours with low, 326 (71.80%) with medium, and 74 (16.30%) with high H-scores. Observer-read and digital assessments were significantly associated (Pearson’s chi-square test, p < 0.001). Higher Trop-2 protein and gene expression were associated with worse distant recurrence-free interval (HR_adj_ = 1.45, 95% CI 1.01–2.08, p = 0.04 and HR_adj_ = 1.33, 95% CI 1.05–1.68, p = 0.02, respectively), particularly in Luminal B subtype.

**Conclusions:**

Trop-2 was detectable across all BC subtypes, exhibiting a wide range of expression levels. Our study suggests that Trop-2 may have a potential prognostic significance in early BC.

## Background

Trophoblast cell surface antigen 2 (Trop-2), a transmembrane glycoprotein [1], has emerged as a compelling tumour-associated cell surface antigen with increased expression across carcinomas [2, 3]. Trop-2 interacts with various signalling pathways and has been associated with tumour development, growth, and metastatic potential [2–6]. In breast cancer (BC), overexpression of Trop-2, particularly in triple-negative breast cancer (TNBC) [4, 7–9], has been linked to poor prognosis [7, 10], making it a critical target for antibody-drug conjugates (ADCs) [11–13].

In the evolving treatment landscape of BC, the first Trop-2–directed ADC, Sacituzumab-Govitecan (SG) [14], has been approved for advanced TNBC [15] and hormone receptor positive/HER2 negative (HR+/HER2−) BC [16]. The clinical efficacy of SG has been demonstrated in several trials, including the phase I/II IMMU 132-01 [17, 18], and the phase 3 ASCENT [19, 20] and TROPiCS-02 trials [21]. Additional Trop-2–directed ADCs under investigation, in the TROPION-Breast01 trial, assessing Datopotamab-Deruxtecan (Dato-DXd) in HR+/HER2− BC [22] and OptiTROP-Breast01, assessing Sacituzumab Tirumotecan (SKB264/MK-2870) in TNBC [23], have shown improved progression-free survival (PFS), supporting these agents as potential therapeutic options for patients with heavily pretreated advanced BC.

In the clinical trials using anti-Trop-2 ADCs, tumour Trop-2 protein expression was not required for study participation, and efficacy was observed irrespective of expression levels [18, 20, 21, 24, 25]. However, target expression remains an important component of the mechanism of action of ADCs, as demonstrated in the DAISY phase II trial, which evaluated the efficacy of Trastuzumab Deruxtecan (T-DXd) in metastatic BC across varying levels of HER2 expression [26]. The apparent lack of predictive value in Trop-2 trials could be due to methodological differences in assessing Trop-2 expression, underscoring the need for standardized evaluation. Although Trop-2 may not directly predict treatment response, its prognostic value could be significant in identifying patients with high-risk tumours who could benefit from Trop-2–directed ADCs.

In this study, we aimed to systematically review and synthesize the current evidence on Trop-2 protein expression and clinical outcomes in patients with BC receiving Trop-2–directed ADCs or chemotherapy. Secondly, we evaluated Trop-2 protein expression in an early BC patient cohort using both observer-read assessment and quantitative image analysis (QIA), as well as Trop-2 gene (TACSTD2) expression, and investigated associations with clinicopathological characteristics and survival outcomes.

## Methods

### Meta-analysis of Trop-2 protein expression: search strategy and study selection

A literature search was performed in Medline, Embase, Cochrane, Web of Science & PubMed, as detailed in the supplementary methods. The initial search was performed in January 2024 and updated in October 2025, using the methods described by Bramer et al. [27]. De-duplication followed Bramer et al. [28]. The full search strategies for all databases are available in Additional file 1: Table S1.

Retrospective cohort studies and randomized clinical trials evaluating Trop-2 protein expression in early or metastatic BC, and the prognostic and/or predictive role (measured as time-to-event outcome) of Trop-2 expression in patients with BC receiving Trop-2–directed ADCs, were included. Two investigators (MT, EL) independently screened studies and extracted data, and a third investigator (PF) cross-checked the databases and resolved discrepancies. The concordance rate was 98%.

Each study’s reporting quality was assessed using the 20-item REMARK (Reporting recommendations for tumour marker prognostic studies) checklist [29]. The study protocol was registered in the International Prospective Register of Systematic Reviews, PROSPERO (CRD42024503618).

### Description of study cohort

The study cohort included patients with primary BC treated between 1997 and 2005 in the health care region of Stockholm-Gotland, Sweden, as previously described [30–32] (Additional file 1: Fig. S1). Distant recurrence-free interval (DRFI) was defined as the time from date of diagnosis to distant metastasis or death from BC and overall survival (OS) as the time from date of diagnosis to death of any cause. Median follow-up was 12.6 years for DRFI and 15.7 years for OS. All cohort analyses were approved by the Swedish Ethical Review Authority (Dnr 2006/394-31/3, 2006/1183-31/2 and amendments 2016/1505-32, 2018/789-32, 2018/790-32, 2021-00113, and 2021-00114).

### Patient tissue samples and immunohistochemistry (IHC) staining

Tissue microarrays (TMAs) were constructed from formalin-fixed paraffin-embedded (FFPE) blocks as previously described [30–32]. Each TMA contained duplicate tumour-rich tissue cores (1 mm diameter). All FFPE sections (4 μm in thickness) were deparaffinized and rehydrated. Heat induced epitope retrieval was performed using Diva decloaker (1:10) in a decloaking chamber (V 4.0.0.0, Biocare Medical, Concord, USA). IHC for Trop-2 utilized a mouse monoclonal anti-Trop-2 antibody (clone: sc-376746, Santa Cruz, Biotechnology, USA, dilution 1:50) and MACH 1 Universal Polymer Detection Kit (BC-M1U539L10). Staining was performed on the intelliPATH FLX - Automated IHC Staining System (Biocare Medical, Concord, USA) according to the manufacturer’s protocol. Positive and negative controls were included (Additional file 1: Fig. S2).

### Scoring of Trop-2 protein expression and quantitative image analysis

Trop-2 protein expression was evaluated using both observer-read assessment and with a digital pathology image analysis software (QuPath), blinded to clinicopathological data, jointly by two trained investigators (MT, ET) and verified by a senior pathologist (GR). Approximately 3% of cases required additional evaluation by the senior pathologist, and consensus was reached for all cases. The expression of Trop-2 was scored in 4 categories according to the overall staining patterns identified: 0 for complete absence of staining, or any staining in ≤10% of tumour cells. 1+ for complete or incomplete membranous staining of low intensity with or without cytoplasmic staining in >10% tumour cells. 2+ for complete or incomplete membranous staining of intermediate intensity with or without cytoplasmic staining in >10% tumour cells. 3+ for complete or incomplete membranous staining of strong intensity with or without cytoplasmic staining in >10% tumour cells.

TMA-stained slides were scanned using Hamamatsu NanoZoomer XR whole-slide scanner (Hamamatsu Photonics K.K., Shizuoka, Japan) at x40 magnification, with a resolution of 0.23 μm/pixel. The files were then loaded onto a project in QuPath software (Source code and documentation for QuPath are available at https://qupath.github.io [33]). TMAs were identified using QuPath’s TMA dearrayer tool and then were manually assessed and verified. Within each TMA core, cells were automatically segmented based on nuclear staining. Cells were then classified using a supervised machine learning approach in which a two-way Random Trees classifier was trained to distinguish tumour cells from stromal and other non-tumour components. Positive cell detection and staining intensity thresholds were optimized using observer-read assessment as ground truth and cross-checked by the senior pathologist. The finalized workflow was applied uniformly across all cores to minimize batch effect and an H-score was calculated for each score by Qu-Path software (see Additional file 1: supplementary methods). Trop-2 protein expression was categorized by QuPath-generated H-score as low (0 to <100), medium (100–200), and high (>200–300) expression.

### Gene expression profiling (GEP)

Total RNA was isolated from primary fresh-frozen tumours using Qiagen RNeasy Mini Kit (Qiagen, Germany). Gene expression profiling was performed using the Rosetta/Merck Human RSTA Custom Affymetrix 2.0 microarray, as previously published [30–32]. Tumour intrinsic subtypes were assigned using the PAM50 classifier with subgroup-specific gene centering [34], as previously detailed [30].

### Statistical methods for the meta-analysis and study cohort analyses

A meta-analysis of proportions was conducted using a random-effects model with inverse-variance weighting to estimate pooled Trop-2 positivity rates and 95% Confidence Intervals (CIs) [35]. To ensure comparability, Trop-2 status (positive vs negative) was defined by grouping levels of 0 and low expression as negative. Subgroup analyses were performed by BC subtype, tumour size, lymph node status, distant metastasis, grade, and histological type. Hazard ratios (HRs) for PFS and overall survival (OS) were extracted from studies comparing Trop-2–directed ADCs with chemotherapy. The meta-analysis was performed according to H-score categories. HRs and their CIs were logarithmically transformed, and a random-effects model was applied to account for heterogeneity. The inverse-variance method was used for weighting, and pooled estimates were back-transformed to the original scale for interpretability. Heterogeneity was assessed using the I^2^ statistic (25, 50, 75% indicating low, moderate and high heterogeneity, respectively) [36]. P-value less than 0.05 was considered statistically significant. All analyses were performed using the Stata statistical software v.14.0.

In the study cohort, associations between Trop-2 protein and clinicopathological characteristics, were examined using the Kruskal-Wallis rank-sum test for continuous variables and Pearson’s chi square test for categorical variables, with Monte Carlo simulated p-values based on 10,000 replicates where appropriate. Associations between Trop-2 gene and clinicopathological characteristics, were examined using Wilcoxon rank-sum test for continuous variables and Fisher’s exact test for categorical variables. The association between observer-read score and QuPath-generated H-score categories was examined using Pearson’s Chi-square test, with standardized residuals. The relationship between continuous QuPath-generated H-score and observer-read score was further assessed using Spearman’s rank correlation, and differences in continuous H-score distributions across observer-read score categories were evaluated using the Kruskal-Wallis rank-sum test. Kaplan-Meier curves estimated DRFI and OS according to Trop-2 protein and gene expression levels. Patients were stratified by observer-read assessment (0–1 vs 2–3), and by digital (QuPath) assessment (low, medium, and high). Univariable and multivariable Cox proportional hazards (PH) regression models estimated HRs and their 95% CIs, with Trop-2 protein expression modelled as a categorical variable (0–1 vs 2–3) for the observer-read assessment and Trop-2 gene expression as a continuous variable. Multivariable models were adjusted for age, tumour size, lymph node status, grade, and proliferation status. Multivariable analyses stratified by IHC-based clinical subtypes (ER+/HER2−, HER2+, ER−/HER2−) and PAM50-based subtypes (Luminal A, Luminal B, HER2-enriched, Basal-like, Normal-like), were adjusted for tumour size, lymph node and proliferation status. Associations between Trop-2 gene and protein expression (observer-read and QuPath) were examined using the Kruskal-Wallis test. Data processing was performed using R programming language (version 4.2.2).

## Results

### Pooled immunohistochemical (IHC) Trop-2 expression

A total of 6394 entries were identified (3610 following deduplication), in the initial search, as shown in the PRISMA Flow chart (Additional file 1: Fig. S3). Upon exclusion based on title and/or abstract screening, 173 possibly eligible studies were retrieved, while 26 records were identified via other methods; 41 studies (32 full text articles and 9 abstracts) fulfilled the inclusion criteria and were included in the meta-analysis [4, 7, 10, 18, 20, 21, 23, 37–70]. The characteristics of eligible studies are presented in Additional file 1: Table S2. The studies had a median reporting quality score of 18 (range: 3 - 32) out of a total of 40.

The overall pooled Trop-2 protein positivity rate was 72% (95% CI, 67–77%; 41 studies, n = 9170), with 79% of TNBC tumours expressing Trop-2 (95% CI, 72–85%; 26 studies, n = 2678). The pooled positivity rates in various subgroups are presented in Table 1.

**Table 1:** Pooled Trop-2 protein positivity rates, overall and in various subgroups

	Number of studies	Trop-2 positive cases (n)	Cases with Trop-2 available data (N)	Effect size (ES), %	95% CI, %	Heterogeneity (I^2^), %	p-value	References
**Overall**	41	9170	12471	72	67–77	98.28	<0.001	[4, 7, 10, 18, 20, 21, 23, 37–70]
**BC Subtype**								
**ER**+**/HER−**	6	480	694	82	67–98	97.52	<0.001	[21, 41, 50, 51, 65, 69]
**HER2**+	3	60	95	70	37–102	94.59	<0.001	[41, 51, 52]
**TNBC**	26	2678	3517	79	72–85	97.23	<0.001	[18, 20, 23, 37–44, 46–49, 51, 58, 59, 62–65, 67–70]
**BC subtype not specified**	21	6115	8358	57	35–79	98.95	<0.001	[4, 7, 10, 40, 42, 43, 45, 47, 48, 50, 51, 53–57, 59–61, 65, 66]
**Tumour Size** ^a^								
**T1**	14	1949	3188	53	33–73	99.57	<0.001	[7, 10, 37, 39, 40, 42, 43, 50, 53, 57, 59, 63, 64, 70]
**T2**	12	1579	2395	67	48–86	99.34	<0.001	[7, 10, 37, 40, 42, 43, 50, 53, 57, 59, 63, 64]
**T3-T4**	12	302	610	54	32–77	98.19	<0.001	[7, 10, 37, 39, 40, 42, 43, 53, 59, 63, 64, 70]
**Lymph Node status** ^b^								
**N0**	13	1950	2735	64	53–74	97.46	<0.001	[7, 10, 37, 39, 40, 42, 43, 50, 53, 59, 63, 64, 70]
**N**+	13	1419	1976	70	59–80	97.29	<0.001	[7, 10, 37, 39, 40, 42, 43, 50, 53, 59, 63, 64, 70]
**Distant Metastasis**								
**M0**	4	244	331	70	54–87	89.79	<0.001	[10, 40, 42, 70]
**M1**	6	396	744	54	39–70	93.25	<0.001	[10, 21, 39, 40, 42, 70]
**Grade**								
**G1**	8	444	817	51	9–93	99.83	<0.001	[7, 10, 37, 39, 50, 53, 57, 70]
**G2**	11	1650	2306	68	52–85	99.05	<0.001	[7, 10, 37, 39, 43, 50, 53, 57, 64, 69, 70]
**G3**	9	1373	1894	70	60–80	95.44	<0.001	[7, 10, 37, 39, 43, 50, 53, 64, 70]
**Histological type**								
**IDC**	12	2334	3469	69	46–93	99.77	<0.001	[7, 10, 39, 40, 45, 47, 50, 53, 59, 63, 64, 69]
**ILC**	6	383	485	62	27–97	98.91	<0.001	[7, 40, 45, 50, 53, 59]
**Other**^c^	12	525	878	57	35–79	98.95	<0.001	[7, 39, 45, 50, 53, 55, 56, 59, 63, 64, 69]

^a^T1, includes tumours ≤2 cm and tumours categorized as T1-2 by the authors; T2, includes tumours >2 cm and ≤5 cm; T3-4, includes tumours >5 cm and tumours categorized as T3-4 by the authors

^b^N+, includes N1, N2, N3

^c^Other: includes medullary breast carcinoma; tubular breast carcinoma; mucinous breast carcinoma; phyllodes breast carcinoma; papillary breast carcinoma; metaplastic breast carcinoma; NEC, breast neuroendocrine carcinoma

*BC* breast cancer, *ER* estrogen receptor, *HER2* human epidermal growth factor receptor, *TNBC* triple negative breast cancer, *IDC* invasive ductal carcinoma, *ILC* invasive lobular carcinoma

### Predictive value of Trop-2 in the meta-analysis

**Fig. 1:**
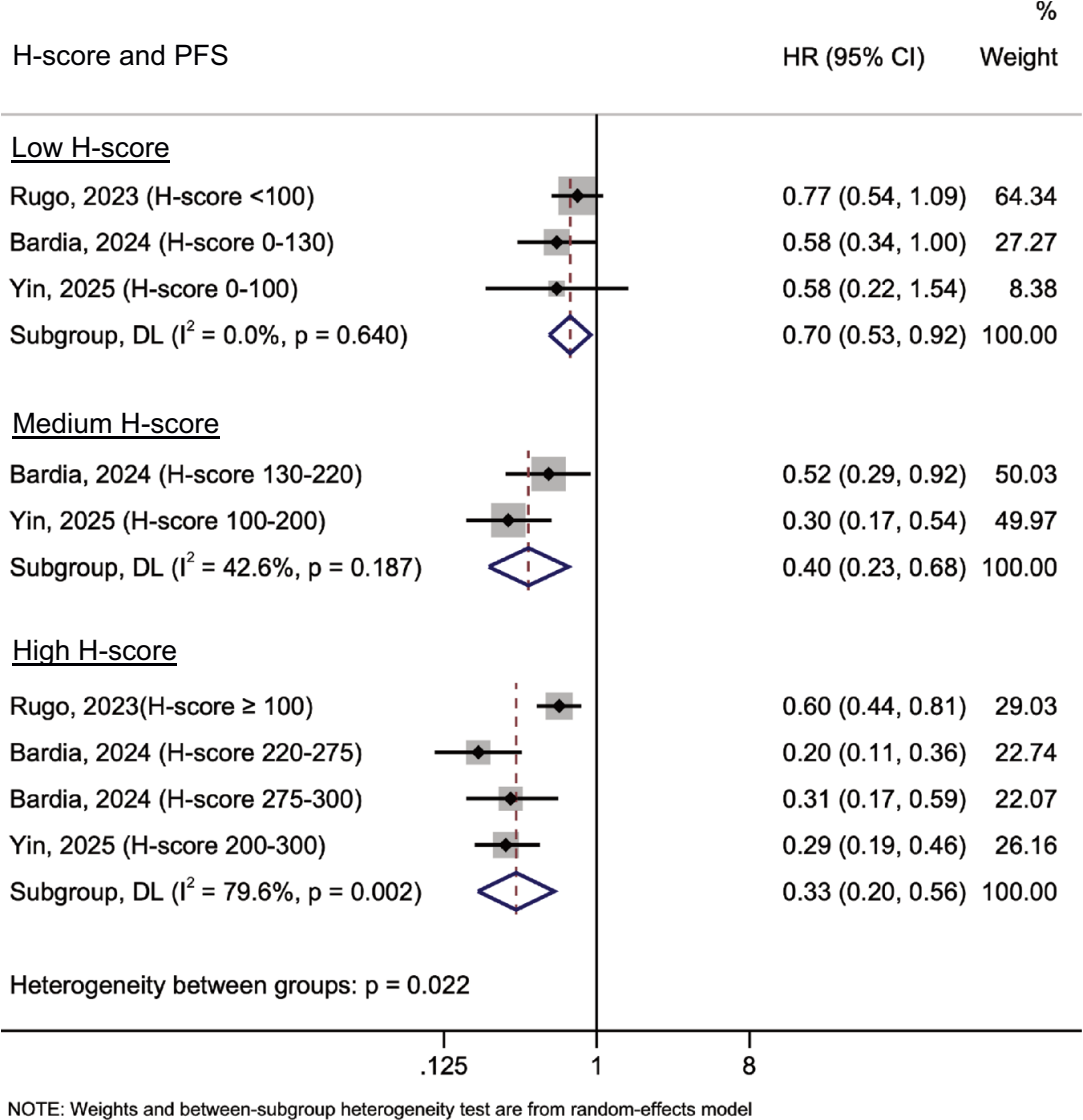
Meta-analysis forest plots of progression-free survival (PFS) according to Trop-2 protein expression by H-score, comparing Trop-2–directed ADCs with chemotherapy. PFS Progression-free survival, ADC Antibody-drug conjugate

The predictive value of Trop-2 protein expression was evaluated in 3 studies across different H-score groups [20, 21, 23]. The overall benefit of Trop-2–directed ADCs compared to chemotherapy in PFS was seen across all H-score groups (Fig. 1). In patients with high H-score, treatment with Trop-2–directed ADC was associated with greater improvement in PFS (3 studies; n = 575; HR = 0.33, 95% CI 0.20–0.56, p < 0.0001). Patients with medium H-score had moderate benefit (3 studies; n = 152; HR = 0.40, 95% CI 0.23–0.68, p = 0.001) and patients with low H-score had the least benefit (3 studies; n = 301; HR = 0.70, 95% CI 0.53–0.92, p = 0.012) (Fig. 1). For OS, a significant benefit was observed only in the low H-score group (2 studies; n = 272; HR = 0.75, 95% CI 0.57–0.98, p = 0.034) (Additional file 1: Fig. S4). No statistically significant improvement in OS was seen in the high H-score group (2 studies; n = 508; HR = 0.62, 95% CI 0.33–1.15, p = 0.128) (Additional file 1: Fig. S4).

### Patient characteristics and histopathological assessment of Trop-2 protein expression in the study cohort

**Fig. 2:**
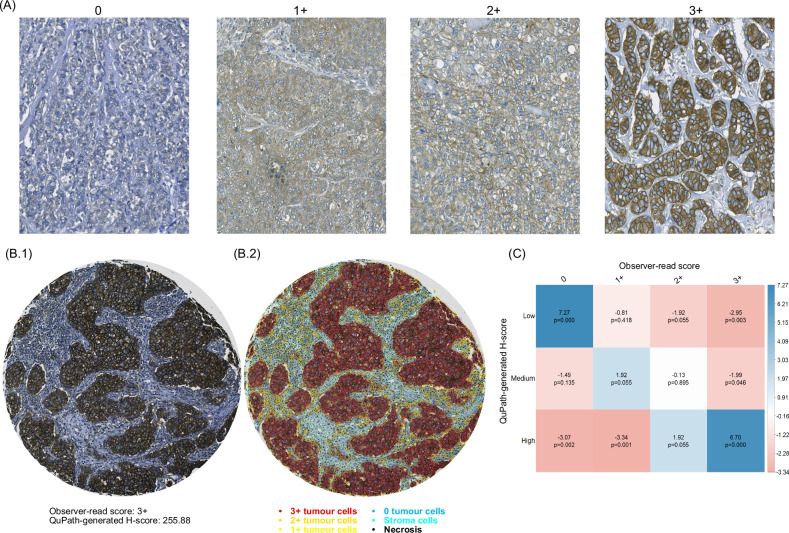
Assessment of Trop‑2 protein expression on tissue microarrays (TMAs) using observer-read assessment and digital image analysis. **A** Expression of Trop-2 on TMAs, with membranous and/or cytoplasmic positivity observed as dark brown colour. 0; zero and 1+; weak Trop-2 expression is shown on the left, 2+; moderate and 3+; high Trop-2 expression is shown on the right (50 μm). **B **Representative images of digital assessment of Trop-2 protein expression with the QuPath Trop-2 cell classifier. (200 μm), (B.1) Observer-read score; 3, (B.2) QuPath-generated H-score; 255.88. **C** Heatmap of standardized Chi-square residuals for the association between observer-read and QuPath assessments of Trop-2 protein expression. Horizontal axis; four categories of observer-read evaluation (0, 1+, 2+, 3+), Vertical axis; three categories of QuPath-generated H-score (low: 0 to <100, medium: 100–200, high: >200-300). Blue cells: category combinations occurring more frequently than expected under independence (positive residuals), Red cells; combinations occurring less frequently than expected (negative residuals) White cells; combinations close to the expected frequency. Colour intensity reflects the magnitude of the standardized residuals

The patients’ clinicopathological characteristics in the cohort study according to Trop-2 protein expression, based on observer-read assessment, are presented in Table 2. In particular, Trop-2 positivity was associated with tumour subtype (<0.001) and QuPath-generated H-score (<0.001). Patient characteristics according to QuPath-generated H-score and Trop-2 gene expression, are presented in Additional file 1: Tables S3 and S4, respectively. Trop-2 staining was evaluated in 454 patients within the cohort study, while 110 patients lacked tumour material on the constructed TMAs. Based on observer-read assessment, Trop-2 protein was expressed in 396 out of 454 patients (87.22%). Specifically, 238 (52.43%) tumours showed 1+ expression, 85 (18.72%) tumours showed 2+, and 73 (16.07%) showed 3+ expression, while 58 (12.78%) tumours had absence of detectable staining (Fig. 2A). Trop-2 protein expression was observed in 20.08% of ER+/HER2− tumours, 81.57% of HER2+ tumours and 35.89% of ER−/HER2− tumours. When Trop-2 protein expression was assessed with quantitative image analysis, 54 (11.90%) tumours displayed low, 326 (71.80%) tumours had medium and 74 (16.30%) high QuPath-generated H-score. Subtype analysis showed that 86.5% of ER+/HER2− tumours, 98.7% of HER2+, and 83.3% of ER−/HER2− tumours had medium or high QuPath-generated H-scores (Fig. 2B). Significant association was observed between observer-read and QuPath score categories of Trop-2 protein expression (Pearson’s chi-square test, p < 0.001), (Fig. 2C). Consistently, continuous QuPath-generated H-score was positively associated with the categorical observer-read score (Spearman’s ρ = 0.617, p < 0.001) (Additional file 1: Fig. S5). When Trop-2 protein and gene were examined, Trop-2 gene expression did not differ significantly across protein expression categories in either observer-read (Kruskal-Wallis, p = 0.90) or QuPath assessment (Kruskal-Wallis, p = 0.08), (Additional file 1: Fig. S6).

**Table 2:** Patient characteristics for all patients in the study cohort and according to Trop-2 protein expression by observer-read assessment

Clinicopathological characteristics	N	0 n = 58^a^	1+ n = 238^a^	2+ n = 85^a^	3+ n = 73^a^	p- value^b^
**Age**	454					0.9
Median (Q1, Q3)		53(46,66)	55(45,63)	53(46,61)	56(48,62)	
**Tumour size**	445					0.13
Median (Q1, Q3)		20(16,29)	20(16,28)	25(17,34)	22(17,28)	
Unknown		0	4	1	4	
**Lymph Node Status**	440					0.11
Positive		26 (46%)	104 (45%)	34 (41%)	21 (30%)	
Negative		31 (54%)	125 (55%)	49 (59%)	50 (70%)	
Unknown		1	9	2	2	
**Grade**	449					0.059
Grade I		4 (6.9%)	24 (10%)	7 (8.3%)	3 (4.2%)	
Grade II		25 (43%)	118 (50%)	35 (42%)	25 (35%)	
Grade III		29 (50%)	93 (40%)	42 (50%)	44 (61%)	
Unknown		0	3	1	1	
**Ki-67**	447					0.10
≤20%		27 (47%)	119 (51%)	37 (44%)	25 (35%)	
>20%		31 (53%)	114 (49%)	47 (56%)	47 (65%)	
Unknown		0	5	1	1	
**IHC subtype**	398					<0.001
ER+/HER2−		32 (67%)	163 (77%)	34 (46%)	15 (23%)	
HER2+		5 (10%)	9 (4.3%)	20 (27%)	42 (65%)	
ER−/HER2−		11 (23%)	39 (18%)	20 (27%)	8 (12%)	
Unknown		10	27	11	8	
**PAM50-based subtype**	443					<0.001
Luminal A		27 (47%)	106 (46%)	29 (35%)	14 (20%)	
Luminal B		14 (25%)	64 (28%)	20 (24%)	12 (17%)	
HER2-enriched		0 (0%)	13 (5.6%)	11 (13%)	29 (41%)	
Basal-like		14 (25%)	43 (19%)	24 (29%)	13 (18%)	
Normal-like		2 (3.5%)	5 (2.2%)	0 (0%)	3 (4.2%)	
Unknown		1	7	1	2	
**QuPath H-score**	454					<0.001
Low (0–100)		26 (45%)	24 (10%)	4 (4.7%)	0 (0%)	
Medium (100–200)		32 (55%)	196 (82%)	60 (71%)	38 (52%)	
High (200–300)		0 (0%)	18 (7.6%)	21 (25%)	35 (48%)	

^a^n (%), percentages calculated based on the number of patients with available data

^b^Kruskal-Wallis rank sum test; Pearson’s Chi-square test with simulated p-value (based on 10000 replicates)

*ER* estrogen receptor, *PR* progesterone receptor, *HER2* human epidermal growth factor receptor 2, *IHC* immunohistochemistry

### Prognostic significance of Trop-2 protein and gene expression in the study cohort

**Fig. 3:**
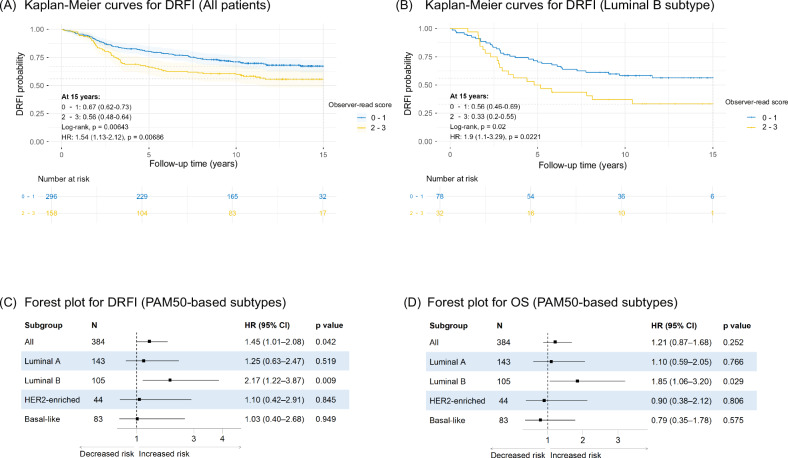
Prognostic significance of Trop‑2 protein expression in the early BC patient cohort. Kaplan–Meier curves for distant recurrence-free interval (DRFI) according to Trop-2 protein expression based on observer-read assessment (0–1 vs 2–3), shown for (**A**) all patients and (**B**) the PAM50-defined Luminal B tumours. Forest plots showing multivariable Cox regression analyses of (**C**) distant recurrence-free interval (DRFI) and (**D**) overall survival (OS) according to Trop-2 protein expression as a categorical biomarker (0–1 vs 2–3), overall and stratified by PAM50-based subtypes. DRFI Distant recurrence-free interval, OS Overall survival, HER2 human epidermal growth factor receptor 2, HR Hazard-ratio, CI Confidence Interval

Trop-2 protein expression, according to observer-read assessment, was significantly associated with worse DRFI, with tumours showing higher expression (scores 2–3 vs 0–1) having poorer outcomes (Log-rank test p = 0.0064) (Fig. 3A), particularly in Luminal B tumours (Log-rank test p = 0.02) (Fig. 3B). However, these associations were not observed when Trop-2 protein expression was assessed digitally using QuPath-generated H-score, either across all subtypes (Log-rank test p = 0.16) or within Luminal B tumours (Log-rank test p = 0.093) (Additional file 1: Fig. S7). The association between Trop-2 protein expression and OS was also examined but was not statistically significant using either observer-read (p = 0.105) or QuPath-based evaluation (p = 0.201), (Additional file 1: Fig. S8). In the multivariable analysis, Trop-2 protein, according to observer-read evaluation, was likewise associated with worse DRFI (adjusted HR (HR_adj_) = 1.45, 95% CI 1.01–2.08, p = 0.04), but not OS (HR_adj_ = 1.21, 95% CI 0.87–1.68, p = 0.25). This effect was primarily driven by Luminal B tumours, with significant associations observed for both DRFI (HR_adj _= 2.17, 95% CI 1.22–3.87, p = 0.009) and OS (HR_adj_ = 1.85, 95% CI 1.06–3.2, p = 0.029) (Fig. 3C, D and Additional file 1: Table S5).

Similarly, in multivariable analysis, higher Trop-2 gene expression was associated with worse DRFI (HR_adj_ = 1.33, 95% CI 1.05–1.68, p = 0.02), but not with OS (HR_adj _= 1.16, 95% CI 0.96–1.4, p = 0.12) (Additional file 1: Table S6). Subgroup analyses revealed worse DRFI across several clinical subtypes, including HER2+ (HR_adj _= 1.7, 95% CI 1.1–2.63, p = 0.02) and ER − /HER2− (HR_adj _= 2.32, 95% CI 1.06–5.09, p = 0.04) tumours. In analyses stratified by PAM50 subtype, worse DRFI was also observed in Luminal B (HR_adj_ = 1.58, 95% CI 1.01–2.46, p = 0.04) and Basal-like (HR_adj_ = 1.81, 95% CI 1.05–3.11, p = 0.03) tumours (Additional file 1: Table S6).

## Discussion

Trop-2 has emerged as an important therapeutic target, as demonstrated by the clinical benefit of Trop‑2–directed ADCs. However, the potential utility of Trop-2 as a biomarker needs to be further studied. Using multiple approaches, we showed that Trop-2 is expressed at varying levels across all BC subtypes in our cohort study, consistent with the published literature [7] and our study-based meta-analysis. This wide range of expression suggests that a broad patient population might be eligible for Trop-2–directed ADCs and supports ongoing investigation of these agents across different BC subtypes and treatment settings [71–73]. Given that target expression is a key component of the mechanism of action of ADCs, variability in Trop-2 expression levels could contribute to heterogeneous responses, and the extent of clinical benefit across different Trop-2 expression levels warrants further investigation.

Heterogeneity in Trop-2 biomarker assessment across studies represents a key challenge and includes differences in whether protein or gene expression is measured and whether observer-read or digital assessment is used for protein evaluation. To our knowledge, this is the first study to examine both Trop-2 protein and gene expression across all BC subtypes within the same patient cohort, using both observer-read and digital quantitative approaches for the protein assessment. The weak correlation between Trop-2 protein and gene expression might be related to post-transcriptional regulatory mechanisms and is consistent with prior reports, suggesting Trop-2 presence in intracellular compartments and complex TACSTD2 regulation [4, 57]. Although Trop-2 is broadly expressed in epithelial tissues, considerable variability in expression levels has been reported across BC subtypes and between normal and malignant tissues [10]. This suggests that differences in Trop-2 expression may reflect underlying tumour biology rather than simply the epithelial origin of the tumour. Despite this, observer-read and digital assessments of Trop-2 protein were significantly correlated, supporting digital pathology as a more reproducible method. However, the discrepancy in prognostic significance between the two methods should be acknowledged. Although the two methods were significantly associated, only the observer-read assessment demonstrated prognostic significance. This may reflect the categorization of continuous H-score values into predefined expression groups, which may not fully preserve the variability of Trop-2 protein expression, particularly for cases near threshold values, as well as differences in the biological relevance of staining patterns and cellular localization captured by each approach. These findings in combination with the heterogenous results of our meta-analysis due to the different scoring methods, highlight the need for a standardized Trop-2 assay, similar to the evolution of HER2 testing and classification. Lastly, the recent development of the AI-based Trop-2 IHC assay in lung cancer demonstrates the feasibility of quantitative scoring and supports our approach using digital pathology in BC [74]. However, further optimization, validation, and standardization of digital Trop-2 assessment in BC are needed.

The clinical significance of target expression extends beyond its potential predictive value and can be influenced by factors such as the interaction between the target and the ADC, as well as certain molecular and histopathological features. As multiple ADCs against different antigens are being developed, the effect of target expression in downstream pathways and surrounding cells may guide therapy selection. Previous studies have highlighted the significant role of Trop-2 in tumour metastasis and promotion of epithelial-mesenchymal transition (EMT) [4, 42], suggesting that higher expression may corelate with more aggressive disease. In our cohort, high Trop-2 expression was associated with worse survival, supporting a potential prognostic role in early BC. Further studies should investigate Trop-2 dynamics during neoadjuvant treatment and how Trop-2 expression changes in residual disease and during tumour progression.

Our meta-analysis confirmed significant benefit from Trop-2–directed ADCs across different Trop-2 expression levels, with greater benefit in tumours with higher Trop-2 expression. However, while a clearer association between Trop-2 expression levels and PFS benefit was observed, the OS findings were less consistent. The apparent discrepancy may reflect the limited number of available studies, differences between the included patient populations, immature OS data, and the potential influence of subsequent therapies on OS outcomes. Thus, the magnitude of benefit based on Trop-2 protein expression levels should be further explored and prospectively validated before Trop-2 expression can be used to guide patient selection or ADC sequencing in clinical practice. Additionally, as these agents are now vigorously evaluated in neoadjuvant and post-neoadjuvant settings [20, 22, 23], Trop-2 expression in primary or residual tumours warrants further investigation, both as prognostic factor to stratify patients at greater risk of relapse and as predictive factor to potentially identify those who could benefit more.

The study has certain limitations that need to be addressed. First, differences in Trop-2 IHC scoring, antibodies, cut-offs, and patient selection contributed to high heterogeneity in our study-based meta-analysis. Additionally, the variability in exposures and outcomes among the studies, precluded further subgroup analyses for prognostic implications. Pre-analytical factors, inter-observer variability, and tumour heterogeneity may have affected the accuracy of Trop-2 IHC assessment. Finally, when adjusting for the three QuPath intensity thresholds, we used the observer-read assessment as the reference, and therefore risk of model overfitting cannot be entirely excluded.

## Conclusions

Trop-2 was detectable across all BC subtypes with a wide range of expression, in both our study cohort and the published literature. This study provides, to our knowledge, the most comprehensive evaluation of Trop-2 protein expression, integrating observer-read and quantitative image analysis, as well as gene expression in the same patient cohort. Our meta-analysis confirmed clinical benefit from Trop-2-directed ADCs across all expression levels, with greater efficacy observed in tumours with higher Trop-2 expression. Taken together, our findings suggest that Trop-2 may serve as a prognostic marker in early BC, and further studies are warranted to clarify its potential clinical utility as a biomarker in early and metastatic BC.

## Supplementary information

Additional file 1 **Supplementary methods**, **Tables S1-S6**, and **Figures S1-S8**. Supplementary methods-Search strategy of Trop-2 protein expression; Description of the QuPath workflow. Table S1-Full search strategies for all databases. Table S2-Characteristics of studies included in the pooled IHC Trop-2 analysis and meta-analysis. Table S3-Patient characteristics by Trop-2 protein expression (QuPath H-score). Table S4-Patient characteristics by Trop-2 gene expression. Table S5-Univariable and multivariable Cox regression analyses of Trop-2 protein expression. Table S6-Univariable and multivariable Cox regression analyses of Trop-2 gene expression. Figure S1-Flow diagram of patient selection for the cohort study. Figure S2-Controls used for immunohistochemistry staining (IHC). Figure S3-PRISMA flowchart. Figure S4-Forest plots for OS by Trop-2 expression: Trop-2–directed ADCs vs chemotherapy. Figure S5-Boxplot of QuPath-generated H-score by observer-read Trop-2 protein expression groups. Figure S6-Boxplots of Trop-2 gene expression by protein expression groups. Figure S7-Kaplan–Meier curves for DRFI by Trop-2 protein expression (QuPath H-score). Figure S8-Kaplan–Meier curves for OS by Trop-2 protein expression (observer-read score and QuPath H-score)

## Data Availability

Data are available upon reasonable request from the corresponding author. Microarray GEP data can be accessed via the Gene Expression Omnibus depository (accession number GSE48091).

## Abbreviations

BC: Breast cancer
QIA: Quantitative image analysis
HR: Hormone receptor
ER: Estrogen receptor
PR: Progesterone receptor
HER2: Human epidermal growth factor receptor 2
Trop-2: Trophoblast cell surface antigen 2
TACSTD2: Tumour-associated calcium transducer 2
TNBC: Triple Negative Breast Cancer
ADC: Antibody-drug conjugate
SG: Sacituzumab Govitecan-hziy
OS: Overall survival
PFS: Progression-free survival
DRFI: Distant recurrence-free interval
IHC: Immunohistochemistry
FFPE: Formalin-fixed paraffin-embedded
TMAs: Tissue microarrays
HIER: Heat induced Epitope Retrieval
GEP: Gene expression profiling
HRs: Hazard Ratios
PH: Proportional Hazards
CΙ: Confidence Interval
T-DXd: Trastuzumab Deruxtecan
EMT: Epithelial-mesenchymal transition
Dato-DXd: Datopotamab-Deruxtecan
SKB264/MK-2870: Sacituzumab Tirumotecan
