# Supplementary material for: Assessment of Trophoblast cell surface antigen 2 (Trop-2) expression and its clinical significance in breast cancer: a multi-level analysis of protein and gene expression
Source: BMC Med. 2026 Jul 31;24:422. doi: 10.1186/s12916-026-05093-3 (PMC13445770; doi:10.1186/s12916-026-05093-3)

Supplementary material to

Assessment of Trophoblast cell surface antigen 2 (Trop-2) expression and its clinical significance in breast cancer: a multi-level analysis of protein and gene expression

Authors: Maria Angeliki Toli* [a], Michail Sarafidis [a], Panagiotis Filis [a,b], Evangelos Tzoras [a], Nikolaos Tsiknakis [a], Emmanouil Sifakis [a], Efstathia Liatsou [c,d], Georgios Rassidakis [a,e,f], Jonas Bergh [a,g], Alexios Matikas [a,g], Ioannis Zerdes [a,h], Theodoros Foukakis [a,g]

Affiliations: [a] Department of Oncology and Pathology, Karolinska Institutet, Stockholm, Sweden; [b] Department of Hygiene and Epidemiology, School of Medicine, University of Ioannina, Ioannina, Greece; [c] Department of Surgery, Sahlgrenska University Hospital, Gothenburg, Sweden; [d] Department of Research, Development, Education and Innovation (FOUUI), Sahlgrenska University Hospital, Gothenburg, Sweden; [e] Department of Clinical Pathology and Cancer Diagnostics, Karolinska University Hospital, Stockholm, Sweden; [f] Department of Hematopathology, The University of Texas MD Anderson Cancer Center, Houston, TX, USA; [g] Breast Center, Karolinska Comprehensive Cancer Center, Karolinska University Hospital, Stockholm, Sweden; [h] Theme Cancer, Karolinska Comprehensive Cancer Center, Karolinska University Hospital, Stockholm, Sweden

*** Corresponding author** (maria.angeliki.toli@ki.se)

Table of contents

Supplementary methods: Search strategy of Trop-2 protein expression 3

Supplementary methods: Description of QuPath workflow 3

Table S1: Full search strategies for all databases 4

Table S2: Characteristics of studies included in pooled IHC Trop-2 analysis and meta-analysis 8

Table S3: Patient characteristics by Trop-2 protein expression (QuPath H-Score) 11

Table S4: Patient characteristics by Trop-2 gene expression 12

Table S5: Univariable and multivariable Cox regression analyses of Trop-2 protein expression 13

Table S6: Univariable and multivariable Cox regression analyses of Trop-2 gene expression 14

Figure S1: Flow diagram of patient selection for the cohort study 15

Figure S2: Controls used for immunohistochemistry staining (IHC) 15

Figure S3: PRISMA flowchart 16

Figure S4: Forest plots for OS by Trop-2 expression: Trop-2–Directed ADCs vs chemotherapy 18

Figure S5: Boxplot of QuPath-generated H-score by observer-read Trop-2 protein expression groups 19

Figure S6: Boxplots of Trop-2 gene expression by protein expression groups 20

Figure S7: Kaplan–Meier curves for DRFI by Trop-2 protein expression (QuPath H-Score) 21

Figure S8: Kaplan–Meier curves for OS by Trop-2 protein expression (observer-read score & QuPath H-Score) 22

Supplementary methods: Search strategy of Trop-2 protein expression

A literature search was performed in the following databases: Medline, Embase, Cochrane, Web of Science & PubMed. The search strategy was developed in Medline (Ovid) in collaboration with librarians at the Karolinska Institutet University Library. For each search concept Medical Subject Headings (MeSH-terms) and free text terms were identified. No language restriction was applied. Databases were searched from inception. The strategies were peer reviewed by another librarian prior to execution. One final, extra step was added to compare DOIs.

Supplementary methods: Description of QuPath workflow

The tissue cores were identified using TMA dearrayer command in QuPath. The resulting TMA grid was manually assessed and verified. Stain and background vectors (QuPath's Estimate stain vectors command) were applied to refine colour deconvolution, while Cell Detection command was performed based on nuclear staining. A two-way Random Trees Classifier was trained interactively using QuPath's Object Classification tool to distinguish tumour cells from stroma and other non-tumour components (i.e. necrosis, immune cells, adipose tissue). The Classifier was applied across all cores, once the training was adequate. Then, the QuPath's Positive Cell Detection command was utilized to classify tumour cells as positive or negative. Additionally, the Threshold Definition parameter was set to further categorize cells according to Trop-2 staining intensity. All parameters were fine-tuned by (MT and ET) and threshold definition used as ground truth the observer-read assessment of Trop-2 staining that was cross-checked with a senior pathologist (GR). An H-score was calculated for each core by the QuPath software by adding 3 × % cells with strong intensity staining, 2 × % cells with moderate intensity staining and 1 × % cells with weak (or mild) intensity staining, giving results in the range 0 (all tumour cells negative) to 300 (all tumour cells positive).

Table S1: Full search strategies for all databases

1. Medline

Interface: Ovid MEDLINE(R) and Epub Ahead of Print, In-Process & Other Non-Indexed Citations and Daily

Date of Search: January, 2024

Number of hits: 950

Comment: In Ovid, two or more words are automatically searched as phrases; i.e. no quotation marks are needed

Field labels

- exp/ = exploded MeSH term
- / = non exploded MeSH term
- .ti,ab,kf. = title, abstract and author keywords
- adjx = within x words, regardless of order
- * = truncation of word for alternate endings

#	Query	Hits	Comments
1	TACSTD2 protein, human.nm.	226	
2	TROP2 protein, mouse.nm.	29	
3	(trop2 or trop-2).ti,ab,kf	561	Terms not truncated to avoid results such as trop$_2$dad, trop$_2$dach
4	(trophoblast* and antigen 2).ti,ab,kf	167	Trophoblast* AND antigen* results in too much noise, as does cell-surface AND antigen 2. Proximity operators do not improve sensitivity compared to AND.
5	(EGP1 or EGP-1).ti,ab,kf	23	
6	(epitheli* and (glycoprotein-1 or glycoprotein1) and antigen*).ti,ab,kf	11	Omitting the glycoprotein part gives too much noise, even when using proximity operators instead of AND.
7	GA733-1.ti,ab,kf	15	No other combination with 733-1 is relevant.
8	733-1.ti,ab,kf	30	
9	M1S1.ti,ab,kf	45	
10	(Chromosome 1 and Surface Marker 1).ti,ab,kf	4	
11	TACSTD2.ti,ab,kf	124	
12	(calcium and transducer 2).ti,ab,kf	65	Calcium AND transducer give too much noise. Using proximity operators does not improve sensitivity.
13	sacituzumab govitecan.ti,ab,kf,nm.	296	On their own, the terms give too much noise. Truncation does not improve sensitivity.
**14**	**or/1-13**	**950**	

2. Embase

Interface: embase.com

Date of Search: January, 2024

Number of hits: 2492

Field labels

- /exp = exploded Emtree term
- /de = non exploded Emtree term
- ti,ab,kw = title, abstract and author keywords
- NEAR/x = within x words, regardless of order
- * = truncation of word for alternate endings

Comment: Emtree is the controlled vocabulary in Embase	

#	Query	Hits	Comments
1	'sacituzumab govitecan'/de	1044	
2	‘trop2 gene’/de	21	All of these are candidate terms and as such have no narrower terms.
3	‘trop2 protein’/de	43	
4	‘tacstd2 gene’/de	46	
5	‘tacstd2 protein, human’/de	23	
6	'trophoblast cell surface antigen 2'/de	115	
7	(trop2 or trop-2):ti,ab,kw	1112	
8	(trophoblast* AND ‘antigen 2’):ti,ab,kw	258	
9	(EGP1 or EGP-1):ti,ab,kw	52	
10	(epitheli* and (glycoprotein-1 or glycoprotein1) and antigen*):ti,ab,kw	17	
11	GA733-1:ti,ab,kw	18	
12	733-1:ti,ab,kw	125	
13	M1S1:ti,ab,kw	256	
14	(‘Chromosome 1’ and ‘Surface Marker 1’):ti,ab,kw	4	
15	TACSTD2:ti,ab,kw	213	
16	(calcium and ‘transducer 2’):ti,ab,kw	94	
17	‘sacituzumab govitecan’:ti,ab,kw	560	
**18**	**or/1-17**	**2494**	

3. Cochrane Library

Interface: Wiley<br><br>Date of Search: January, 2024<br><br>Number of hits: 201	Field labels<br><br>• ti,ab,kw = title, abstract and author keywords<br>• NEAR/x = within x words, regardless of order<br>• * = truncation of word for alternate endings

ID	Search	Hits
#1	(trop2 or trop-2):ti,ab,kw	101
#2	(trophoblast* and "antigen 2"):ti,ab,kw	17
#3	(EGP1 or EGP-1):ti,ab,kw	5
#4	((glycoprotein-1 or glycoprotein1) and antigen* and epithel*):ti,ab,kw	0
#5	("733-1"):ti,ab,kw	39
#6	("GA733-1"):ti,ab,kw	0
#7	(M1S1):ti,ab,kw	8
#8	("Chromosome 1" and "Surface Marker 1"):ti,ab,kw	0
#9	(TACSTD2):ti,ab,kw	1
#10	(calcium and "transducer 2"):ti,ab,kw	2
#11	("sacituzumab govitecan"):ti,ab,kw	116
#12	(OR #1-#11)	202
#13	#12 in Trials	201

4. Web of Science Core Collection

Interface: Clarivate Analytics<br><br>Editions = A&HCI , ESCI , SCI-EXPANDED , SSCI<br><br>Date of Search: January, 2024<br><br>Number of hits: 1562	Field labels<br><br>• TS/Topic = title, abstract, author keywords and Keywords Plus<br>• NEAR/x = within x words, regardless of order<br>• * = truncation of word for alternate endings<br><br>Note: Sometimes “quotation marks” are needed for single search terms to avoid automatic term mapping (lemmatization).

#	**Query**	**Results**
1	TS=(trop2 or trop-2)	820
2	TS=(trophoblast* and "antigen 2")	180
3	TS=(EGP1 or EGP-1)	39
4	TS=(epitheli* and (glycoprotein-1 or glycoprotein1) and antigen*)	12
5	TS=(GA733-1)	15
6	TS=(733-1)	121
7	TS=(M1S1)	52
8	TS=("Chromosome 1" and "Surface Marker 1")	4
9	TS=(TACSTD2)	124
10	TS=(calcium and "transducer 2")	59
11	TS=("sacituzumab govitecan")	667
12	OR/1-11	1562

5. PMC

Interface: NCBI<br><br>Date of Search: January, 2024<br><br>Number of hits: 1189<br><br>Comment: In PMC, when searching in specific fields, two or more words are automatically searched as phrases; i.e. no quotation marks are needed	Field labels<br><br>• [text word] – searches in the entire reference as well as the article body<br>• [title], [abstract] – in PMC one cannot search for title and abstract simultaneously<br>• [Supplementary Concept] – chemical or substance names<br><br>• * – truncation of word for alternate endings<br>• OR/#1-#x – shorthand for #1 OR #3 OR … OR #x Cannot be entered directly in PMC.

Search	Query	Items found
#40	#39 OR #17	1189
#39	#26 AND #38	890
#38	#27 OR #28 OR #29 OR #30 OR #31 OR #32 OR #33 OR #34 OR #35 OR #36 OR #37	4755
#37	Search sacituzumab govitecan[text word]	1304
#36	Search (calcium[text word] AND transducer 2[text word])	600
#35	Search TACSTD2[text word]	1134
#34	Search (Chromosome 1[text word] AND Surface Marker 1[text word])	18
#33	Search M1S1[text word]	214
#32	Search 733-1[text word]	324
#31	Search GA733-1[text word]	56

#30	Search (epitheli*[text word] AND (glycoprotein-1[text word] OR glycoprotein1[text word]) AND (antigen*[text word] OR antigens[text word]))	177
#29	Search (EGP1[text word] or EGP-1[text word])	151
#28	Search (trophoblast[text word] AND antigen 2[text word]))	774
#27	Search (trop2[text word] or trop-2[text word])	2451
#26	#18 OR #25	162388
#25	#21 AND #24	155585
#24	#22 OR #23	1328319
#23	Search (cancer*[title] OR cancer*[abstract] OR carcinoma*[title] OR carcinoma*[abstract] OR neoplas*[title] OR neoplas*[abstract] OR tumor*[title] OR tumor*[abstract] OR tumour*[title] OR tumour*[abstract])	1181390
#22	Search neoplasms[mesh]	676128
#21	#19 OR #20	194863
#20	Search (breast*[title] OR breast*[abstract] OR lobular[title] OR lobular[abstract] OR mammary[title] OR mammary[abstract] OR nipple*[title] OR nipple*[abstract] OR paget*[title] OR paget*[abstract])	194220
#19	Search breast[MeSH Terms]	8848
#18	Search breast neoplasms[MeSH Terms]	77588
#17	#1 OR #2 OR #3 OR #4 OR #5 OR #6 OR #7 OR #8 OR #9 OR #10 OR #11 OR #12 OR #13 OR #14 OR #15 OR #16	418
#16	Search (sacituzumab govitecan[title] OR sacituzumab govitecan[abstract])	129
#15	Search ((calcium[title] AND transducer 2[title]) OR (calcium[abstract] AND transducer 2[abstract]))	37
#14	Search ((calcium[title] and transducer 2[title]) OR (calcium[abstract] and transducer 2[abstract]))	0
#13	Search (TACSTD2[title] OR TACSTD2[abstract])	67
#12	Search ((Chromosome 1[title] AND Surface Marker 1[title]) OR (Chromosome 1[abstract] AND Surface Marker 1[abstract]))	1
#11	Search ((Chromosome 1[title] and Surface Marker 1[title]) OR (Chromosome 1[abstract] and Surface Marker 1[abstract]))	0
#10	Search (M1S1[title] OR M1S1[abstract])	9
#9	Search (733-1[title] OR 733-1[abstract])	4
#8	Search (GA733-1[title] OR GA733-1[abstract])	3
#7	Search ((epitheli*[title] and (glycoprotein-1[title] or glycoprotein1[title]) and (antigen*[title] OR antigens[title])) OR (epitheli*[abstract] and (glycoprotein-1[abstract] or glycoprotein1[abstract]) and (antigen*[abstract] OR antigens[abstract])))	1
#6	Search (epitheli*[title] and (glycoprotein-1[title] or glycoprotein1[title]) and (antigen*[title] OR antigens[title])) OR (epitheli*[abstract] and (glycoprotein-1[abstract] or glycoprotein1[abstract]) and (antigen*[abstract] OR antigens[abstract])))	1
#5	Search (EGP1[title] or EGP-1[title] OR EGP1[abstract] or EGP-1[abstract])	5
#4	Search ((trophoblast*[title] and antigen 2[title]) OR (trophoblast*[abstract] and antigen 2[abstract]))	0
#3	Search (trop2[title] or trop-2[title] OR trop2[abstract] or trop-2[abstract])	272
#2	Search TROP2 protein, mouse[Supplementary Concept]	20
#1	Search TACSTD2 protein, human[Supplementary Concept]	91

Table S2: Characteristics of studies included in pooled IHC Trop-2 analysis and meta-analysis

Author, year (reference)	Country	Type of study	Tissue used	Antibody used	Threshold of positivity	Follow-up, months	Quality assessment (REMARK)
Trerotola, 2013 (4)	Italy	Retrospective	TMA	AF650	NA	NA	3
Ambrogi, 2014 (7)	Italy	Retrospective	TMA	162-46.2 (HB-187), AF650	>0 (intensity and % of stained cells)	96	27
Lin, 2013 (10)	China	Retrospective	TMA	NA	>0 (intensity and % of stained cells)	NA	18
Bardia, 2017 (18)	USA	Retrospective	FFPE	NA	>0 or >10%	16.600000000000001	16
Bardia, 2024 (20)	USA, Belgium, UK, Spain, Germany, France, Canada	Retrospective analysis of prospective study	FFPE	ENZ ABS380-0100	H-score >0	17.7	25
Rugo, 2023 (21)	USA, Canada, Europe	Prospective	FFPE	SP295	H-score ≥100	12	32
Yin, 2025 (23)	China	Retrospective analysis of prospective study	NA	EPR20043	H-score >200	10.4	28
Bueno, 2024 (37)	Madrid, Spain	Retrospective	TMA	ABS380	H-score ≥1.48	67.599999999999994	17
Spring, 2024 (38)	USA	Prospective	FFPE	AF650	H-score: above mean value	18.899999999999999	26
Jeon, 2022 (39)	South Korea	Retrospective	TMA	EPR20043	H-score: above mean value	128 (cohort 1), 84 (cohort 2), 26 (cohort 3)	21

Sayama, 2021 (40)	Japan	Retrospective	FFPE	TrMab-6	NA	NA	5
Aslan, 2021 (41)	USA, Hungary	Retrospective	TMA	B9 sc-376746	>0 (intensity)	NA	4
Zhao, 2018 (42)	China	Retrospective	TMA	ab227689	H-score >130	60	15
Ibrahim, 2021 (43)	Egypt	Retrospective	FFPE	2-TU022-13	>0 (intensity and % of stained cells)	36	17
Garrido-Castro, 2023 (44)	USA, UK	Retrospective	NA	NA	H-score >0	NA	NA
Zhao, 2023 (45)	USA	Retrospective	FFPE, TMA	EPR20043	H-score >0	NA	10
Khoury, 2019 (46)	USA	Retrospective	FFPE	NA	≥10%, 1+	NA	NA
Petroni, 2012 (47)	Italy	Retrospective	NA	NA	NA	72	NA
Yang, 2021 (48)	USA	Retrospective	FFPE	ERP20043	H-score >0	NA	NA
Izci, 2022 (49)	Belgium	Retrospective	FFPE	Ab227689	H-score: >0	115.2	28
Coelho, 2023 (50)	Brasil	Retrospective	TMA	B9 sc-376746	H-score >0	NA	22
Mertens, 2024 (51)	USA	Retrospective	FFPE	NA	H-score >0	NA	19
Gion, 2024 (52)	Spain	Retrospective	TMA	SP293,Abcam	H-score 0,10,50	42	32
Dum, 2022 (53)	Germany	Retrospective	FFPE	MSVA-733R	>0 (intensity and % of stained cells)	43	12
Guerra, 2023 (54)	Italy	Retrospective	FFPE	162-46.2 (HB-187), AF650	NA	NA	9
Vranic, 2019 (55)	USA	Retrospective	FFPE	NA	≥ 10%, 2+ intensity	NA	7
Chartier, 2023 (56)	France	Retrospective	FFPE	AF650	≥ 1%	52	15
Guerra, 2013 (57)	Italy	Retrospective	TMA	162-46.2	NA	96	5
Yin, 2024 (58)	China, USA	Retrospective analysis of prospective study	NA	NA	H-score >200	9.6	NA

Liu, 2022 (59)	China	Retrospective	TMA	Clone01	>4 (intensity and % of stained cells)	80	19
Bobos, 2008 (60)	Greece	Retrospective	TMA	162-46	NA	NA	NA
Choi, 2024 (61)	South Korea	Retrospective	TMA	HPA055 067	H-score >0	NA	NA
Cursano, 2025 (62)	Italy	Retrospective	FFPE	Clone B-9 sc-376746	H-score >0	NA	12
Ebrahim, 2025 (63)	Egypt	Retrospective	FFPE	Clone B9	>0 (intensity and % of stained cells)	90.7	22
Jacot, 2025 (64)	France	Retrospective	TMA	ENZ-ABS380	H-score >0	116.4	27
Lai, 2024 (65)	China	Retrospective	FFPE	SP295	>1 +	NA	22
LeVee, 2025 (66)	USA	Retrospective	FFPE	EPR200 43	NA	NA	21
Sun, 2025 (67)	China	Retrospective	TMA	EPR200 43	>0 (intensity)	NA	10
Todo, 2025 (68)	Spain	Retrospective	NA	EPR200 43	H-score ≥100	NA	NA
Van Bockstal, 2024 (69)	Belgiu m	Retrospective	FFPE	BSB-148, EPR200 43, SP293	>1 +	NA	14
Yuceer, 2025 (70)	Turkey	Retrospective	FFPE	Clone B-9	Above negative/l ow (0–3 points)	56.8	18

Table S3: Patient characteristics for all patients in study cohort according to Trop-2 protein expression, based on QuPath evaluation. Protein expression was evaluated according to QuPath-generated H-score (low: 0 to <100 vs medium: 100 to 200 vs high: >200 to 300).

Clinicopathological characteristics	N	Low n=54[1]	Medium n=326[1]	High n=74[1]	p-value[2]
**Age**	454				>0.9
Median (Q1, Q3)		54 (43,66)	54 (46, 63)	55 (47, 61)	
**Tumour size**	445				0.12
Median (Q1, Q3)		20 (17,28)	23 (17, 30)	21 (15, 26)	
Unknown		0	8	1	
**Lymph Node Status**	440				0.3
Positive		27 (51%)	131 (42%)	27 (38%)	
Negative		26 (49%)	184 (58%)	45 (63%)	
Unknown		1	11	2	
Unknown		6	34	8	
**Tumour grade**	449				0.14
Grade I		3 (5.6%)	30 (9.3%)	5 (6.9%)	
Grade II		31 (57%)	146 (45%)	26 (36%)	
Grade III		20 (37%)	147 (46%)	41 (57%)	
Unknown		0	3	2	
**Ki-67**	447				0.2
≤ 20%		31 (57%)	146 (46%)	31 (42%)	
>20%		23 (43%)	174 (54%)	42 (58%)	
Unknown		0	6	1	
**IHC subtype**	398				<0.001
ER+/HER2-		33 (70%)	184 (65%)	27 (41%)	
HER2+		1 (2.1%)	43 (15%)	32 (48%)	
ER-/HER2-		13 (28%)	58 (20%)	7 (11%)	
Unknown		7	41	8	
**PAM50-based subtype**	443				<0.001
Luminal A		27 (52%)	128 (40%)	21 (29%)	
Luminal B		12 (23%)	82 (26%)	16 (22%)	
HER2-enriched		1 (1.9%)	28 (8.8%)	24 (33%)	
Basal-like		12 (23%)	71 (22%)	11 (15%)	
Normal-like		0 (0%)	9 (2.8%)	1 (1.4%)	
Unknown		2	8	1	
**Observer-read score**	454				<0.001
0		26 (48%)	32 (9.8%)	0 (0%)	
1+		24 (44%)	196 (60%)	18 (24%)	
2+		4 (7.4%)	60 (18%)	21 (28%)	
3+		0 (0%)	38 (12%)	35 (47%)	

[1]n (%), percentages calculated based on the number of patients with available data

[2]Kruskal-Wallis rank sum test; Pearson's Chi-square test with simulated p-value (based on 10000 replicates)

Abbreviation: ER, estrogen receptor; PR, progesterone receptor, HER2, human epidermal growth factor receptor 2; IHC, immunohistochemistry.

Table S4: Patient characteristics for all patients in study cohort according to Trop-2 gene expression. Upper quartile was used as a cut-off.

Clinicopathological characteristics	N	Low n=423[1]	High n=141[1]	p-value[2]
**Age**	564			0.2
Median (Q1, Q3)		54 (45,62)	56 (47,63)	
**Tumour size**	552			0.4
Median (Q1, Q3)		22 (16,30)	20 (15,27)	
Unknown		11	1	
**Lymph Node Status**	547			>0.9
Positive		176 (43%)	58 (43%)	
Negative		235 (57%)	78 (57%)	
Unknown		12	5	
**Tumour grade**	535			0.004
Grade I		29 (7.2%)	17 (13%)	
Grade II		175 (43%)	69 (53%)	
Grade III		200 (50%)	45 (34%)	
Unknown		19	10	
**Ki-67**	519			0.13
≤ 20%		171 (44%)	66 (52%)	
>20%		220 (56%)	62 (48%)	
Unknown		32	13	
**IHC subtype**	473			0.3
ER+/HER2-		218 (60%)	65 (58%)	
HER2+		77 (21%)	20 (18%)	
ER-/HER2-		66 (18%)	27 (24%)	
Unknown		62	29	
**PAM50-based subtype**	551			0.001
Luminal A		153 (37%)	62 (45%)	
Luminal B		115 (28%)	24 (17%)	
HER2-enriched		58 (14%)	9 (6.5%)	
Basal-like		76 (18%)	41 (29%)	
Normal-like		10 (2.4%)	3 (2.2%)	
Unknown		11	2	

[1] n (%), percentages calculated based on the number of patients with available data
[2] Wilcoxon rank sum test; Fisher's exact test
Abbreviations: ER, estrogen receptor; PR, progesterone receptor, HER2, human epidermal growth factor receptor 2; IHC, immunohistochemistry

Table S5: Univariable and multivariable Cox regression analyses of distant recurrence-free interval (DRFI) and overall survival (OS) using Trop-2 protein expression as a categorical biomarker (0 – 1 vs. 2 – 3), overall and stratified by IHC-based clinical and PAM50-based subtypes.

Trop-2 protein expression														
Variable	N	Distant Recurrence-Free Interval						Overall Survival					
		Univariable analysis			Multivariable analysis			Univariable analysis			Multivariable analysis		
		HR	95% CI	p-value	HR	95% CI	p-value	HR	95% CI	p-value	HR	95% CI	p-value
**All patients**	384	1.41	1 - 1.99	0.05	1.45	1.01 - 2.08	0.04	1.17	0.86 - 1.6	0.32	1.21	0.87 - 1.68	0.25
**ER+/HER2-**	235	1.1	0.66 - 1.83	0.72	1	0.6 - 1.69	0.99	1.04	0.66 - 1.66	0.85	0.97	0.6 - 1.55	0.89
**HER2+**	71	2.02	0.71 - 5.73	0.19	2.49	0.86 - 7.23	0.09	1.46	0.61 - 3.5	0.4	1.8	0.74 - 4.38	0.2
**ER-/HER2-**	78	1.4	0.52 - 3.77	0.5	1.42	0.52 - 3.89	0.5	0.82	0.35 - 1.92	0.65	0.82	0.35 - 1.94	0.65
**Luminal A**	143	1.39	0.72 - 2.68	0.32	1.25	0.63 - 2.47	0.52	1.14	0.63 - 2.07	0.67	1.1	0.59 - 2.05	0.77
**Luminal B**	105	1.89	1.08 - 3.29	0.03	2.17	1.22 - 3.87	0.01	1.65	0.97 - 2.79	0.06	1.85	1.06 - 3.2	0.03
**HER2-enriched**	44	0.86	0.33 - 2.26	0.76	1.1	0.42 - 2.91	0.85	0.75	0.32 - 1.75	0.5	0.9	0.38 - 2.12	0.81
**Basal-like**	83	1.17	0.46 - 2.98	0.73	1.03	0.4 - 2.68	0.95	0.89	0.4 - 1.96	0.77	0.79	0.35 - 1.78	0.57
**Normal-like**	9	0	0 - Inf	1	0	0 - Inf	1	0	0 - Inf	1	0	0 - Inf	1

N, Number of patients included in the analysis; HR, hazard ratio; CI, confidence interval; ER, estrogen receptor; HER2, human epidermal growth factor receptor 2; IHC, immunohistochemistry.

Table S6: Univariable and multivariable Cox regression analyses of distant recurrence-free interval (DRFI) and overall survival (OS) using Trop-2 (TACSTD2) gene expression as a continuous biomarker, overall and stratified by IHC-based clinical and PAM50-based subtypes.

Trop-2 gene (TACSTD2) expression														
**Variable**	**N**	**Distant Recurrence-Free Interval**						**Overall Survival**						
		**Univariable analysis**			**Multivariable analysis**			**Univariable analysis**			**Multivariable analysis**			
		**HR**	**95% CI**	**p-value**	**HR**	**95% CI**	**p-value**	**HR**	**95% CI**	**p-value**	**HR**	**95% CI**	**p-value**
**All patients**	446	1.24	1 - 1.54	0.05	1.33	1.05 - 1.68	0.02	1.09	0.91 - 1.31	0.34	1.16	0.96 - 1.4	0.12
**ER+/HER2-**	269	1.07	0.81 - 1.4	0.65	1.13	0.85 - 1.49	0.4	0.97	0.77 - 1.22	0.79	1.01	0.8 - 1.27	0.95
**HER2+**	88	1.39	0.98 - 1.98	0.07	1.7	1.1 - 2.63	0.02	1.2	0.85 - 1.69	0.3	1.34	0.92 - 1.97	0.13
**ER-/HER2-**	89	2.38	1.07 - 5.26	0.03	2.32	1.06 - 5.09	0.04	1.77	0.96 - 3.23	0.07	1.69	0.93 - 3.04	0.08
**Luminal A**	164	0.85	0.51 - 1.43	0.55	0.84	0.49 - 1.43	0.52	0.94	0.61 - 1.46	0.79	0.93	0.59 - 1.46	0.75
**Luminal B**	123	1.65	1.07 - 2.57	0.03	1.58	1.01 - 2.46	0.04	1.23	0.88 - 1.71	0.24	1.17	0.84 - 1.63	0.36
**HER2-enriched**	55	1.55	0.87 - 2.76	0.14	1.64	0.88 - 3.03	0.12	1.14	0.73 - 1.78	0.57	1.13	0.7 - 1.82	0.62
**Basal-like**	94	1.78	1.08 - 2.92	0.02	1.81	1.05 - 3.11	0.03	1.53	1.02 - 2.31	0.04	1.51	0.99 - 2.31	0.06
**Normal-like**	10	0.11	0 - 7.25	0.3	0	0 - Inf	1	0.04	0 - 1.52	0.08	0	0 - Inf	0.25

N, Number of patients included in the analysis; HR, hazard ratio; CI, confidence interval; ER, estrogen receptor; HER2, human epidermal growth factor receptor 2; IHC, immunohistochemistry.

Figure S1: Flow diagram of patient selection for the cohort study

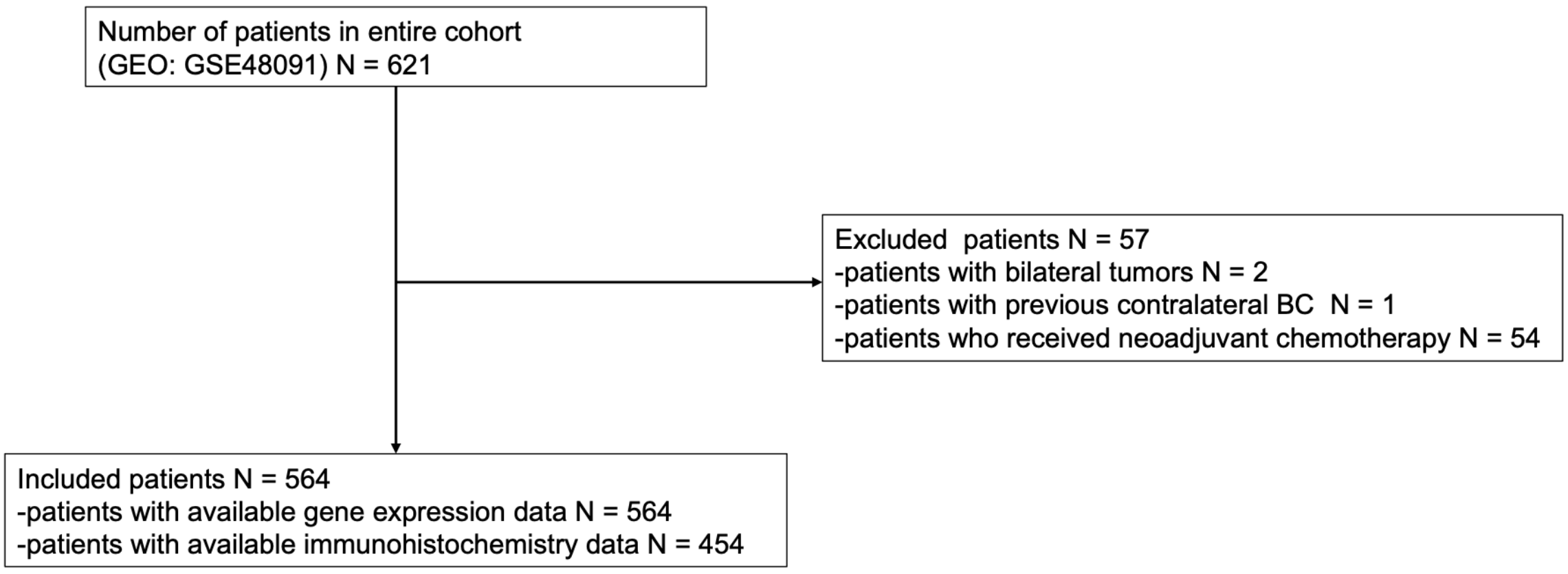

Figure S2: Controls used for immunohistochemistry staining (IHC)

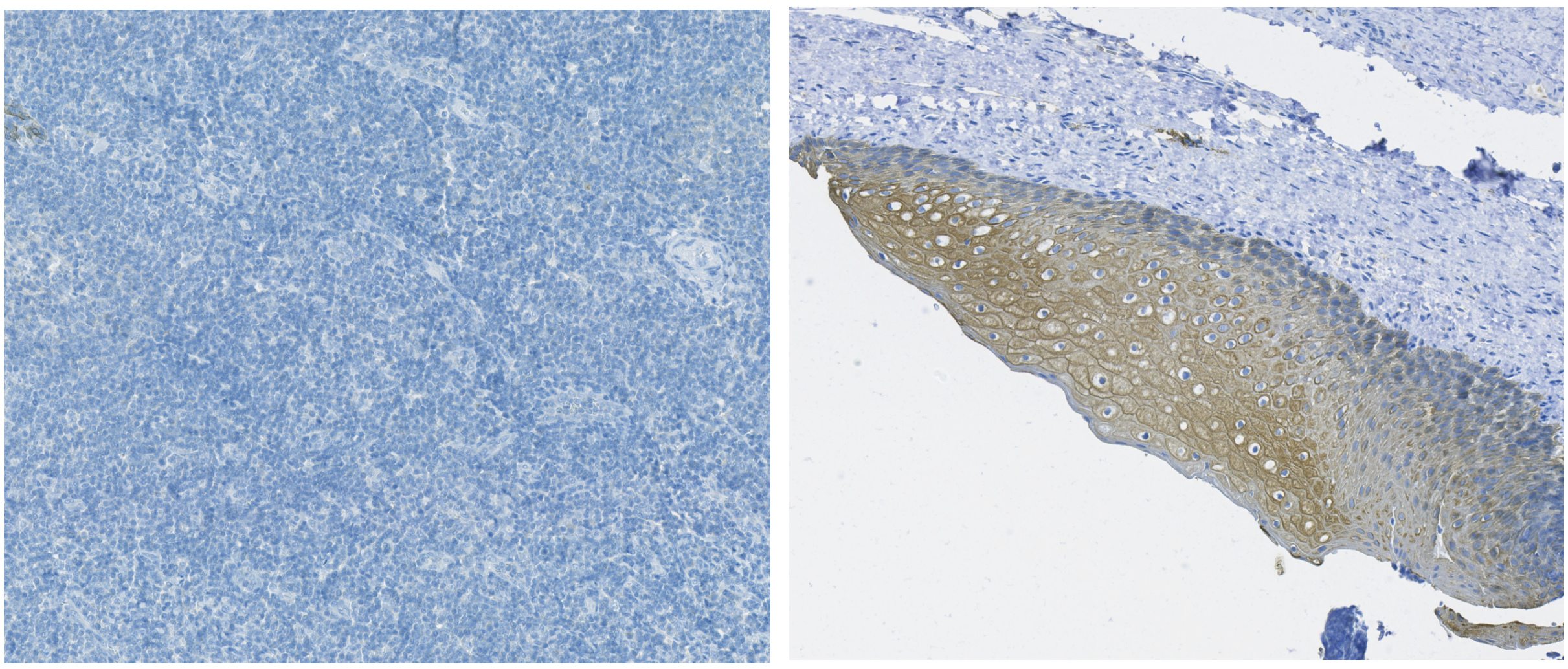

Representative images of tonsil, used as both negative and positive control (15 μm). On the left, tonsil lymphoid tissue is negatively stained for Trop-2. On the right, tonsil squamous epithelium is positively stained for Trop-2.

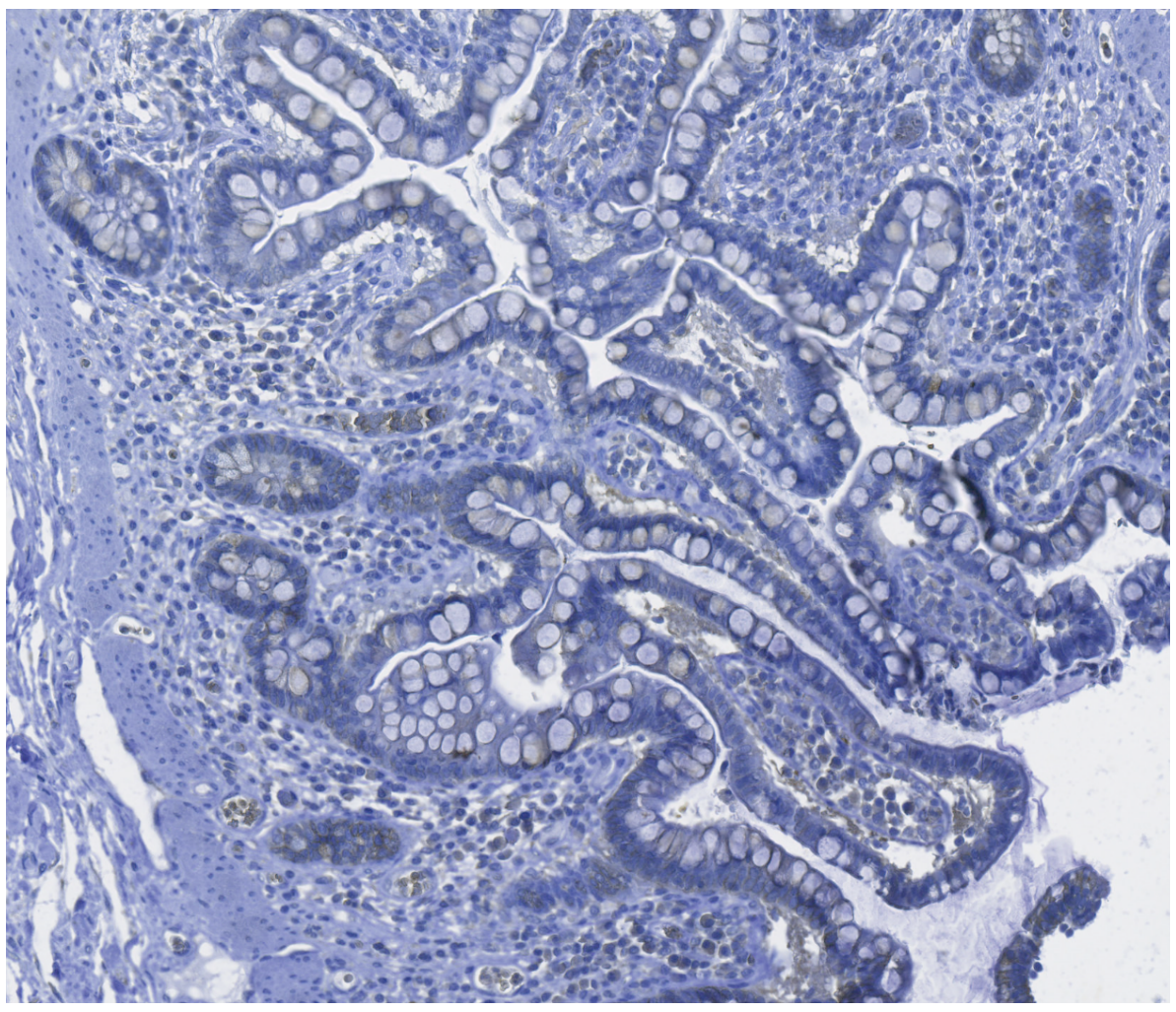

Representative image of intestinal epithelium from colon, used as negative control (15 µm).

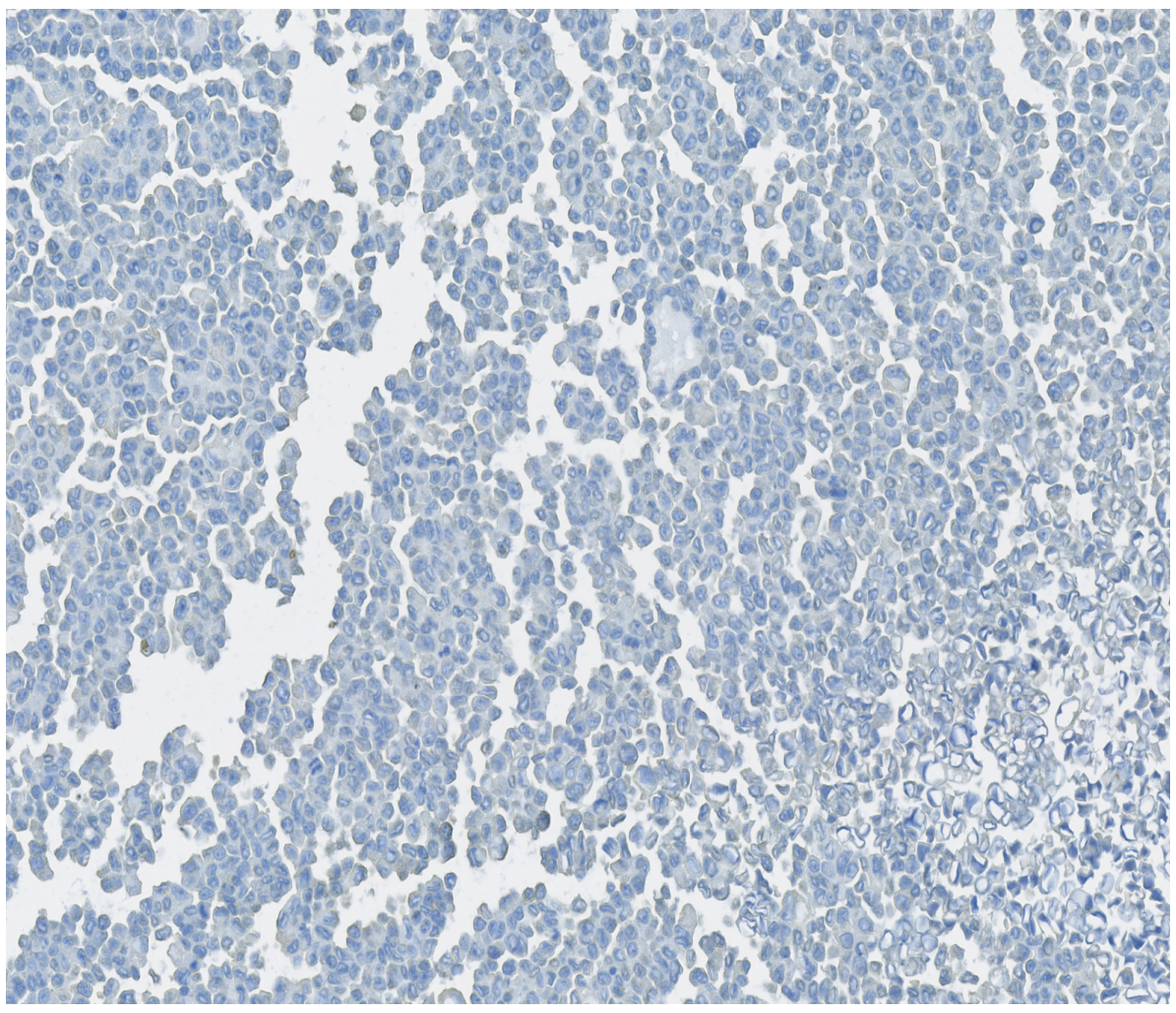

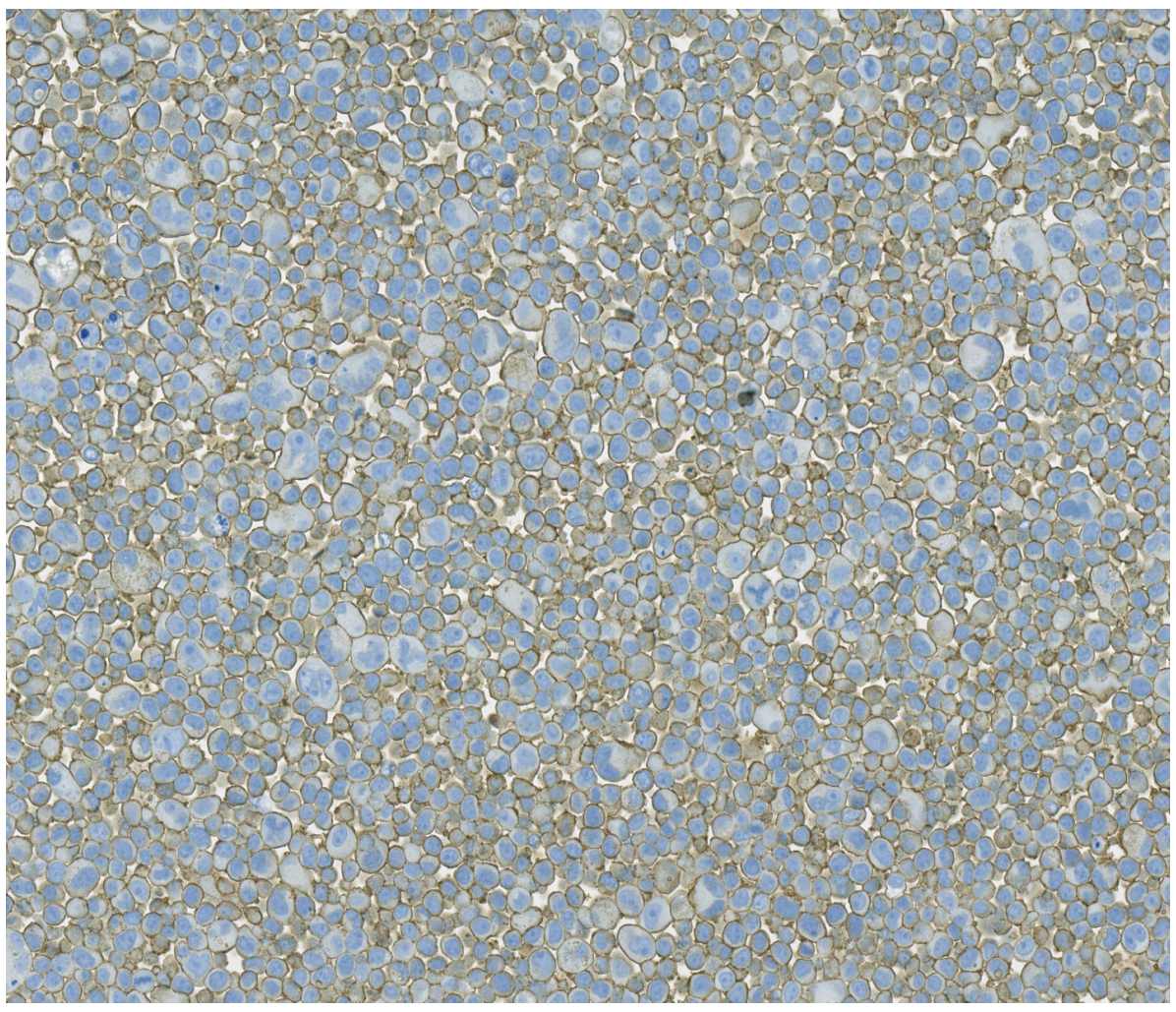

Representative images of breast cancer cell lines used as negative and positive controls (15 µm). On the left, MCF cell line is negatively stained for Trop-2. On the right, BT474 cell is positively stained for Trop-2.

Figure S3: PRISMA flowchart

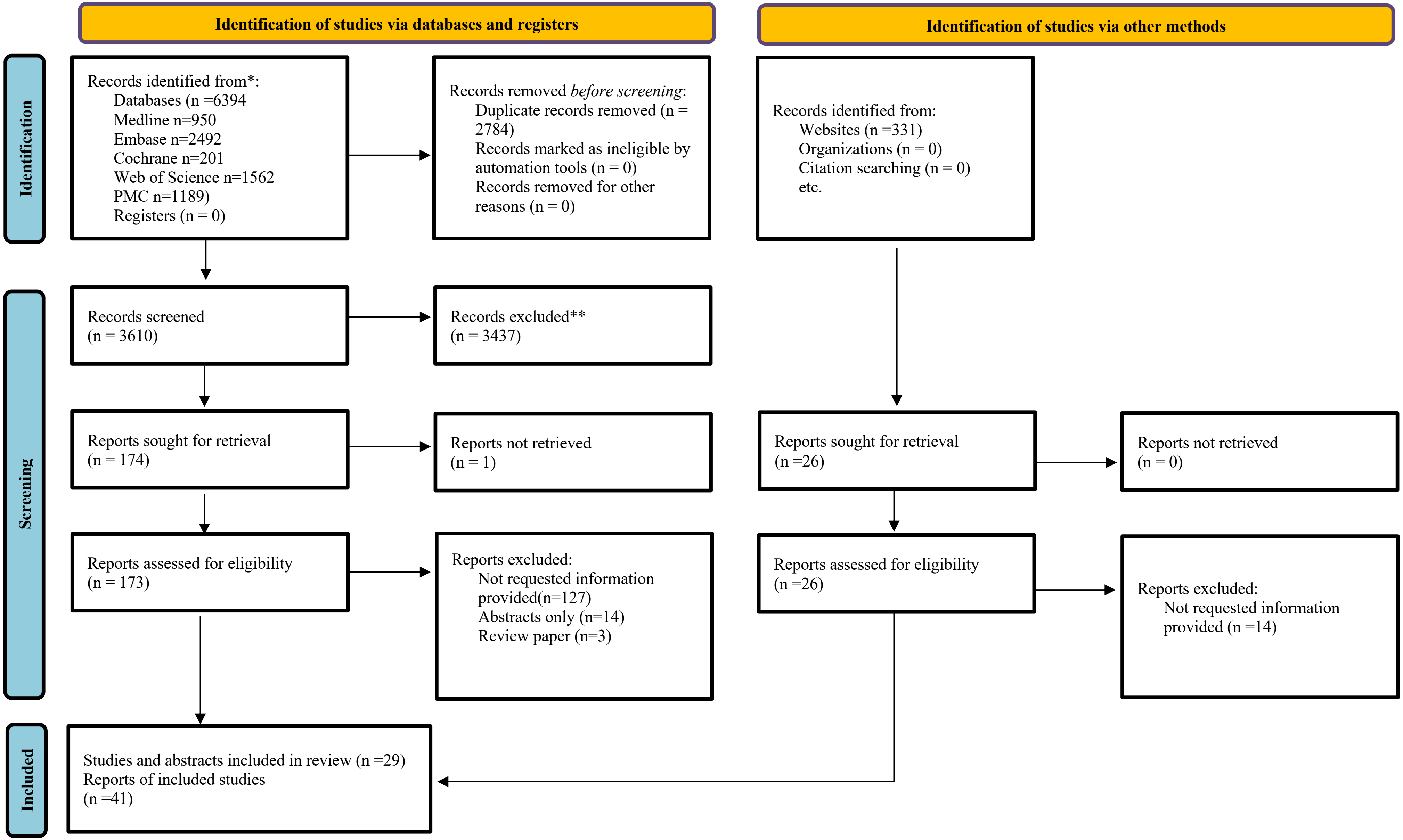

*Consider, if feasible to do so, reporting the number of records identified from each database or register searched (rather than the total number across all databases/registers).
**If automation tools were used, indicate how many records were excluded by a human and how many were excluded by automation tools.

From: Page MJ, McKenzie JE, Bossuyt PM, Boutron I, Hoffmann TC, Mulrow CD, et al. The PRISMA 2020 statement: an updated guideline for reporting systematic reviews. BMJ 2021;372:n71. doi: 10.1136/bmj.n71. For more information, visit: http://www.prisma-statement.org/

Figure S4: Forest plots for overall survival (OS) according to Trop-2 protein expression by H-score comparing Trop-2 directed ADCs to chemotherapy.

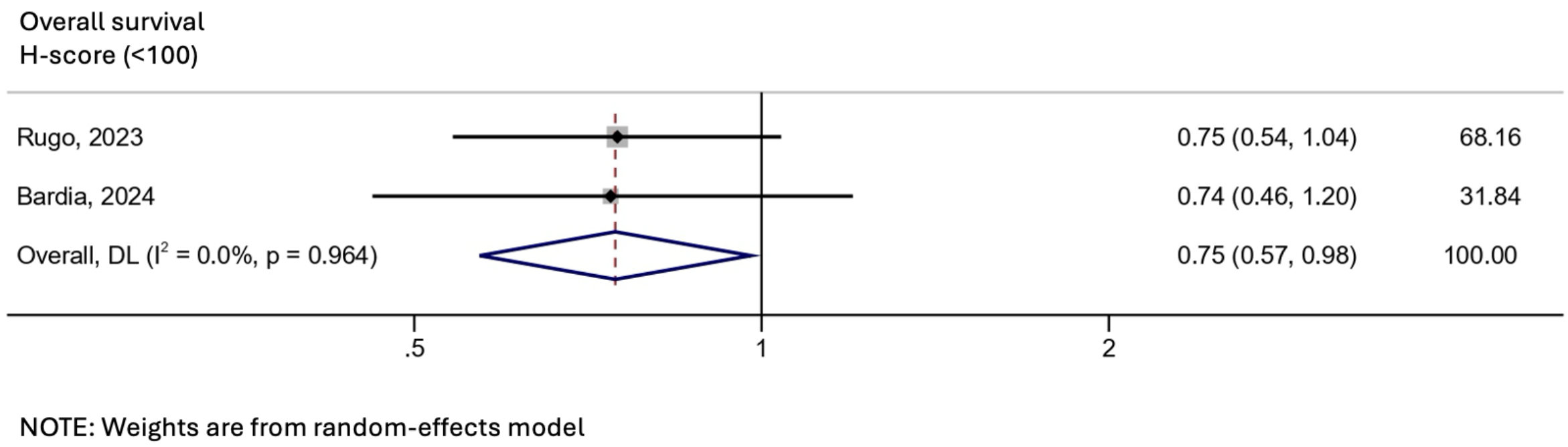

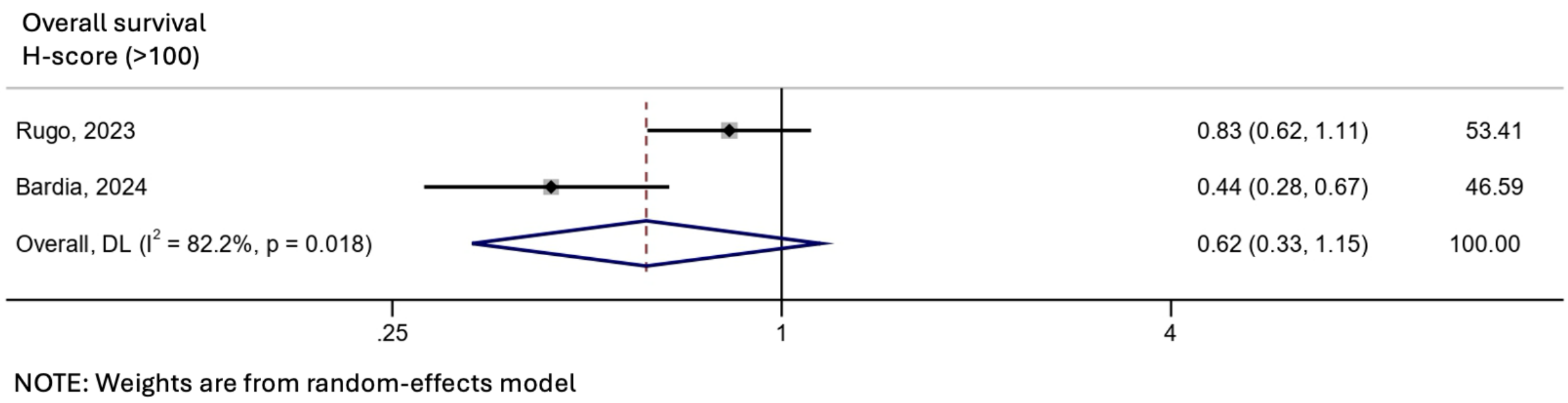

Figure S5: Association between observer-read and QuPath-generated Trop-2 protein expression scores. Boxplots show the distribution of QuPath-generated H-scores across observer-read Trop-2 protein expression categories (scores 0, 1, 2, and 3). The association between observer-read scores and QuPath-generated H-scores was evaluated using Spearman's rank correlation, and differences in H-score distributions between groups were assessed using the Kruskal–Wallis test.

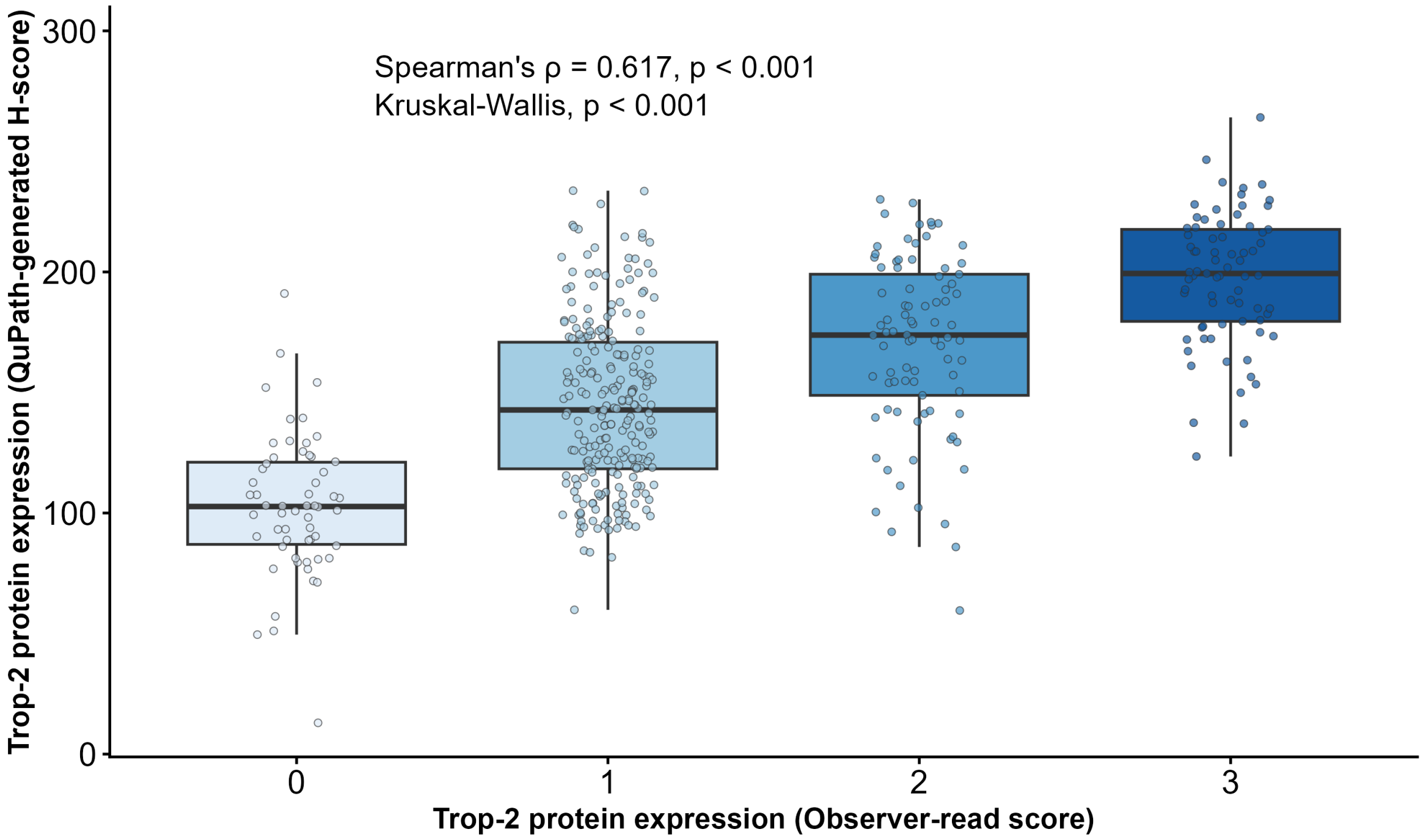

Figure S6: Association between Trop-2 gene expression and protein expression groups. Boxplots show the distribution of Trop-2 gene expression across protein expression categories defined by (A) observer-read score (scores 0, 1, 2, and 3) and (B) QuPath-generated H-score (Low, Medium, and High). Differences in gene expression distributions between groups were evaluated using the Kruskal-Wallis test.

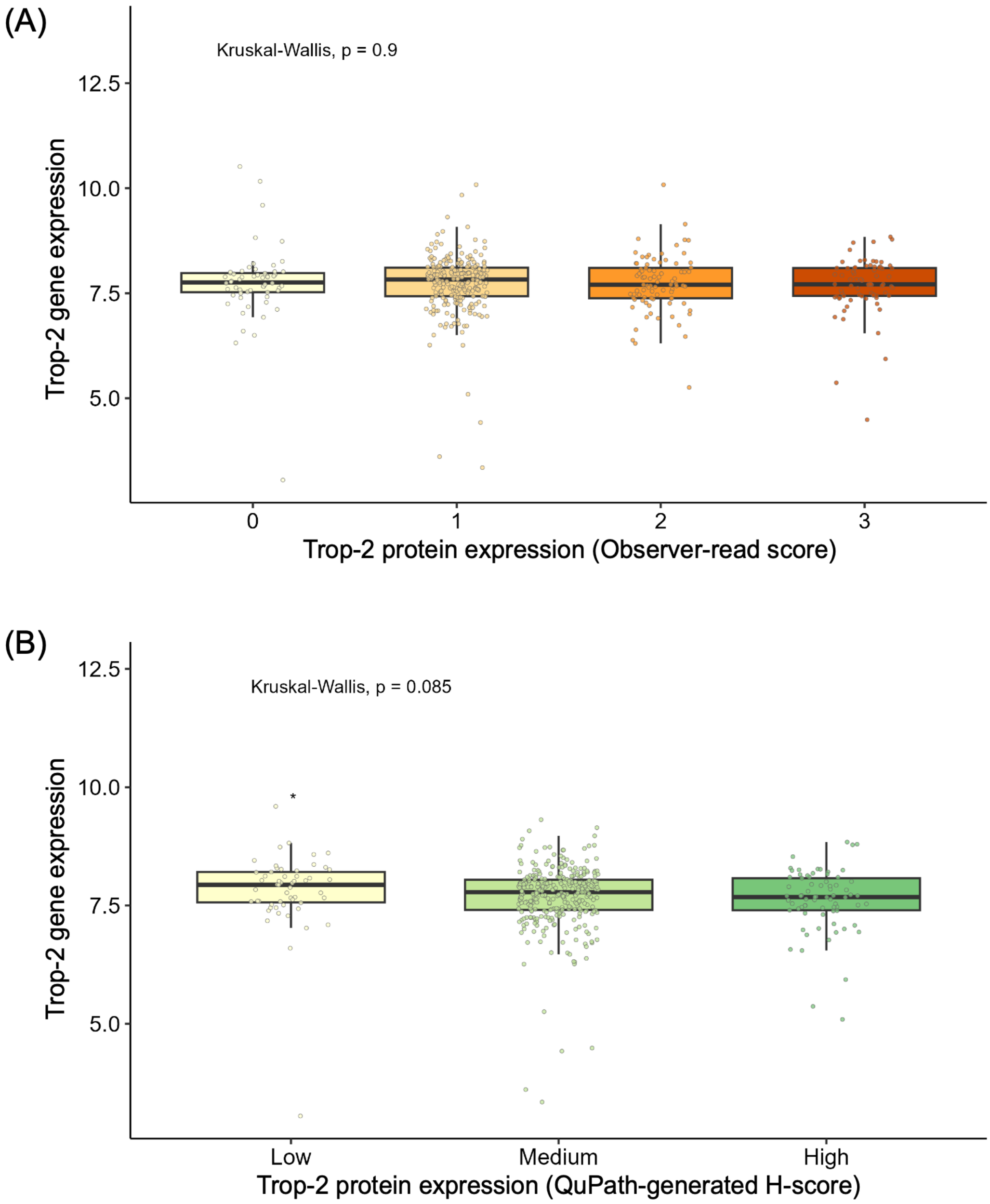

Figure S7: Kaplan-Meier curves for distant recurrence-free interval (DRFI) according to Trop-2 protein expression based on the QuPath-generated H-score (low: 0 to <100, medium: 100–200, and high: >200–300), shown for (A) all clinical subtypes and (B) the PAM50-defined Luminal B subtype.

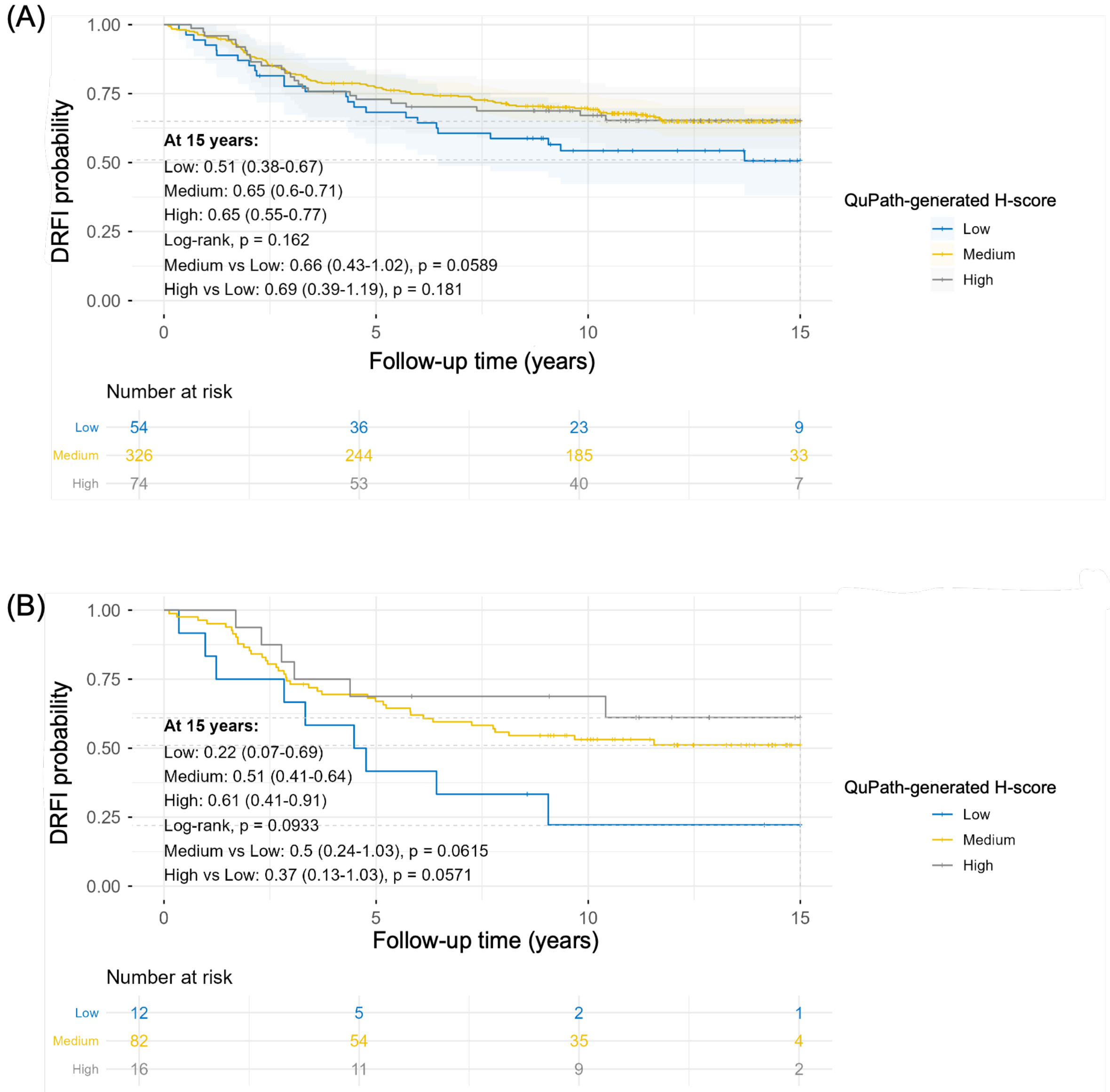

Figure S8: Kaplan-Meier curves for overall survival (OS) according to Trop-2 protein expression based on (A) observer-read score (0-1 vs 2-3) and (B) QuPath-generated H-score (low: 0 to <100; medium: 100–200; high: >200–300) across all clinical subtypes.

(A)

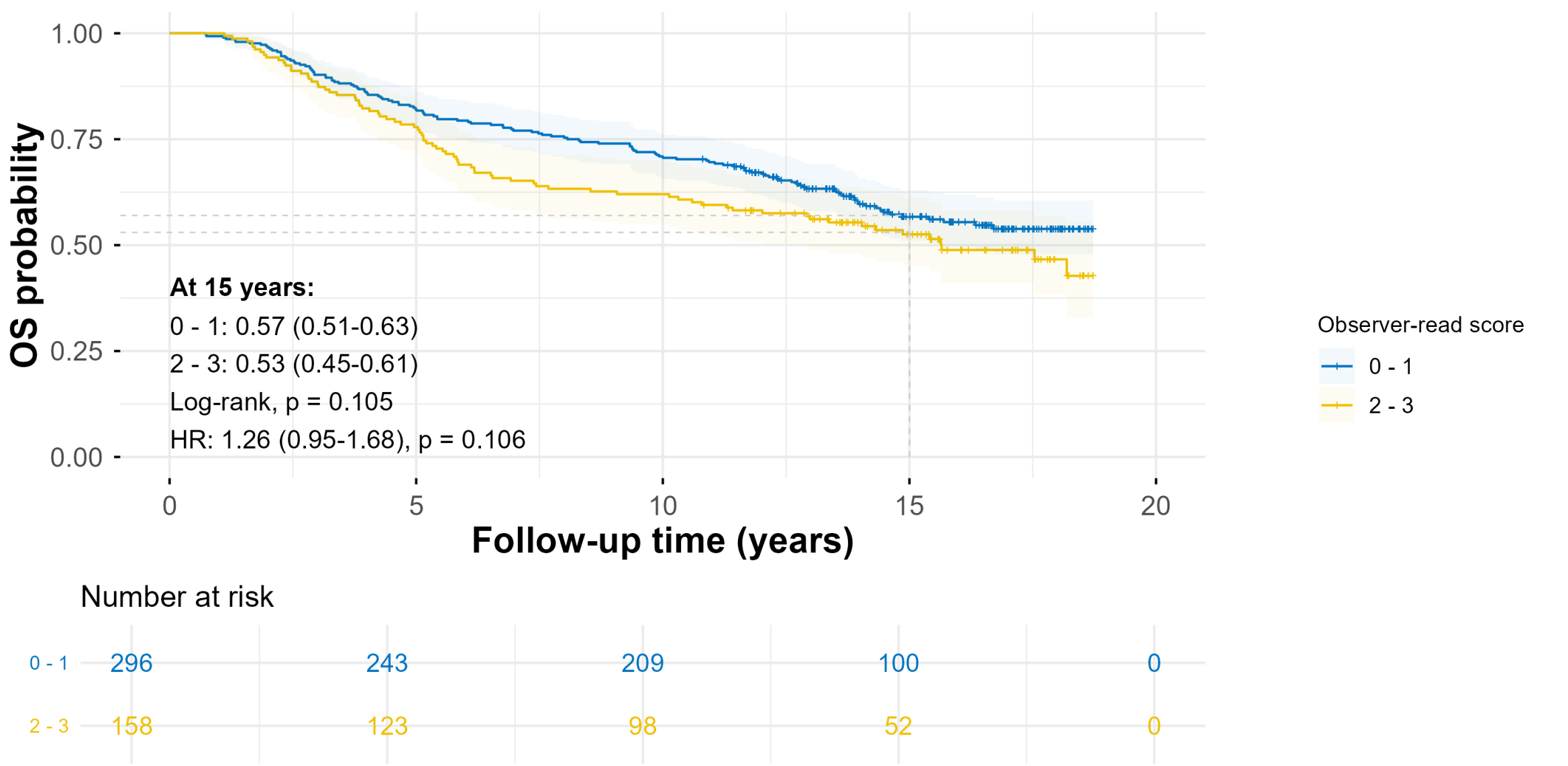

(B)

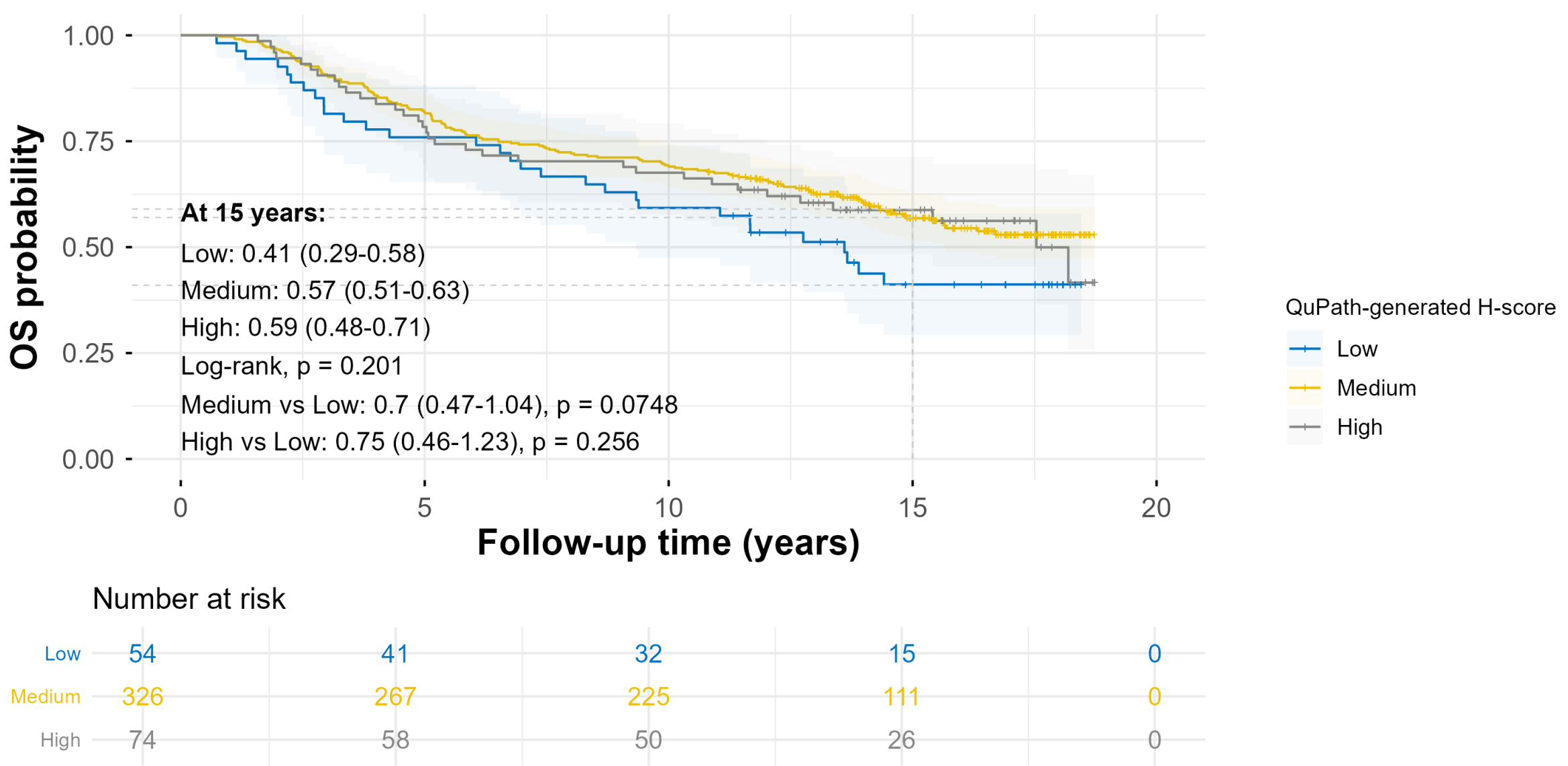

Supplement: Supplementary file 1 [file 12916_2026_5093_MOESM1_ESM.pdf]
